# Altered structural connectivity networks in a mouse model of complete and partial dysgenesis of the corpus callosum

**DOI:** 10.1016/j.neuroimage.2020.116868

**Published:** 2020-04-29

**Authors:** Timothy J. Edwards, Laura R. Fenlon, Ryan J. Dean, Jens Bunt, IRC5 Consortium, Elliott H. Sherr, Linda J. Richards

**Affiliations:** a Queensland Brain Institute, The University of Queensland, St. Lucia, Brisbane, Australia; b Faculty of Medicine, The University of Queensland, Herston, Brisbane, Australia; c Researchers of the International Research Consortium for the Corpus Callosum and Cerebral Connectivity (IRC5); d Department of Neurology, UCSF Benioff Children’s Hospital, San Francisco, CA, USA; e School of Biomedical Sciences, The University of Queensland, St. Lucia, Brisbane, Australia

**Keywords:** Corpus callosum, Corpus callosum dysgenesis, Structural connectome, Cortical development, Brain plasticity, Neurodevelopmental disorders

## Abstract

Corpus callosum dysgenesis (CCD) describes a collection of brain malformations in which the main fiber tract connecting the two hemispheres is either absent (complete CCD, or ‘agenesis of the corpus callosum’) or reduced in size (partial CCD). Humans with these neurodevelopmental disorders have a wide range of cognitive outcomes, including seemingly preserved features of interhemispheric communication in some cases. However, the structural substrates that could underlie this variability in outcome remain to be fully elucidated. Here, for the first time, we characterize the global brain connectivity of a mouse model of complete and partial CCD. We demonstrate features of structural brain connectivity that model those predicted in humans with CCD, including Probst bundles in complete CCD and heterotopic sigmoidal connections in partial CCD. Crucially, we also histologically validate the recently predicted ectopic sigmoid bundle present in humans with partial CCD, validating the utility of this mouse model for fine anatomical studies of this disorder. Taken together, this work describes a mouse model of altered structural connectivity in variable severity CCD and forms a foundation for future studies investigating the function and mechanisms of development of plastic tracts in developmental disorders of brain connectivity.

## 1. Introduction

The corpus callosum (CC) is the largest white matter tract in the mammalian brain. It comprises spatially organized axonal projections that facilitate the bilateral integration of motor, sensory, and associative processes between the two cerebral hemispheres. Individuals born without a full CC (termed corpus callosum dysgenesis, CCD, or agenesis of the corpus callosum) display a broad range of cognitive outcomes and may be on the autistic spectrum (Brown and Paul, 2019; Paul et al., 2014; Paul et al., 2007). Notably, functions that involve the bilateral integration of information between hemispheres remain largely intact in CCD; a clear contrast to individuals who have had a callosotomy later in life (Paul et al., 2007). This has led many to question how structural connections are organized in the absence of callosal fibers. For instance, putative callosal axons are redirected anteroposteriorly in most cases of CCD to form connections within the ipsilateral hemisphere (Probst, 1901; Ren et al., 2007; Utsunomiya et al., 2006). Moreover, structural connectivity studies in small cohorts of humans with CCD using diffusion MRI-based tractography have described ectopic homotopic structural connections between bilateral parieto-occipital cortices in CCD that may contribute to the preservation of interhemispheric functional connectivity (Tovar-Moll et al., 2014). Tractography in individuals who possess a callosal remnant (partial CCD) has further demonstrated considerable variability in the combination of brain regions that this diminished structure connects. Amongst these connections is the sigmoid bundle, an aberrant white matter tract that has been described in a minority of partial CCD individuals, which asymmetrically connects the frontal lobe to the contralateral parieto-occipital cortex via the CC remnant (Tovar-Moll et al., 2007; Wahl et al., 2009; Bénézit et al., 2015). The presence of a sigmoid bundle has been correlated with augmented coherence of EEG signal between the putatively connected regions, indicating that it may mediate functional connectivity (Lazarev et al., 2016). Paradoxically, the anomalous tracts that constitute the proposed core features of structural connectivity in human CCD have only been identified in a minority of cases. This is likely due to a combination of factors, not limited to: genetic heterogeneity, environmental influences and methodological challenges of studying *in vivo* structural connectivity in humans.

Animal models have historically been used to address some of these challenges, as they present an opportunity to study brain connectivity in a more controlled system. However, while the incidence and nature of aberrant structural connectivity has been studied in humans, it has not been fully characterized in mouse models (Olavarria and Van Sluyters, 1995; Dodero et al., 2013; Vega-Pons et al., 2017). Very few cases of partial CCD in mice have been described, which has to date precluded the development of a mouse model to study aberrant connectivity through the callosal remnant in partial CCD (Edwards et al., 2014). The establishment of such a mouse model is critical to replicate and validate analogous connectivity changes to those in humans in a system where histological tract tracing is possible, and therefore to aid development of predictive diagnoses and therapeutic interventions.

The purpose of the present study was to conduct a systematic investigation and comparison of changes in connectivity in mouse models of both complete and partial CCD. To this end, we backcrossed wild type C57Bl/6J mice twice to complete CCD BTBR T+ tf/J (BTBR) mice to generate BTBR x C57Bl/6 N2 (BTBR N2) littermates that display either full corpus callosum, complete or partial CCD (Jones-Davis et al., 2013). High resolution *ex vivo* diffusion MRI (dMRI) and tractography was employed to mathematically model the axonal connections between spatially separated cortical regions; these connectivity maps were in turn used to reconstruct the topological organization of the large-scale brain networks (or connectomes) found in both complete and partial CCD mice. These networks were subsequently compared by utilizing whole network graph theory measures and network-based statistics (NBS) to identify key differences between callosal conditions (Zalesky et al., 2010). By applying methods similar to those previously used to characterize human CCD to a mouse strain with CCD, differences in structural connectivity were identified that resemble those observed in humans. Crucially, we were able to validate our tractography findings by *in utero* electroporation and immunohistochemistry, providing strong evidence for structural developmental plasticity in the mouse CCD brain. Our findings establish a mouse line with variable degrees of callosal malformations as a model for investigating the variability, plasticity and function of changes in structural connectivity across humans and mice with CCD.

## 2. Materials and methods

### 2.1. Animals

Breeding and experimental protocols were approved by The University of Queensland Animal Ethics Committee and were performed according to the Australian Code of Practice for the Care and Use of Animals for Scientific Purposes. Mice used in this study were generated by reciprocal crosses between BTBR and C57BL/6J inbred mouse strains. F1 females were crossed with BTBR males to generate littermates (BTBR N2) with varying degrees of CCD. For all MR imaging, at postnatal days (P) 80–82, mice were anesthetized and transcardially perfused with 0.9% saline, followed by 4% paraformaldehyde (PFA). Brains were postfixed in 4% PFA for at least 48 h before storage in phosphate buffered saline (PBS) with 0.2% sodium azide. Brains were dissected from the skull and incubated in PBS with 0.2% gadopentetate dimeglumine (Magnevist, Berlex Imaging, Wayne, NJ, USA) for four days prior to imaging. To further characterize the neuroanatomy of the BTBR N2 mouse (Jones--Davis et al., 2013), prior to full brain structural imaging, each brain was scanned using a single diffusion direction to identify the callosal phenotype. In total, corpus callosum length was measured in n = 112 animals from 13 litters; of these, 10 brains of each callosal phenotype (complete CCD, partial CCD and normal CC) were scanned utilizing the full scanning protocol described below.

### 2.2. Data acquisition

dMRI volumes were acquired for complete CCD, partial CCD, and normal CC mouse brains (n = 10 for each condition). Prior to scanning, each brain was immersed in Fomblin Y-VLAC oil (Y06/6 grade, Solvay, USA); air was actively removed from each sample via vacuum pumping. Scanning was conducted using a 16.4 T vertical bore, small animal MRI system (Bruker Biospin, Rheinstetten, Germany; ParaVision v5.0) equipped with Micro 2.5 imaging gradient and a 15 mm linear, surface acoustic wave coil (M2M, Brisbane, Australia). Parameters for the dMRI spin-echo pulse sequence were as follows: matrix size (MTX) 196 × 114 × 84, field of view (FOV) 19.6 × 11.4 × 8.4 mm (0.10 mm isotropic voxels), repetition time/echo time (TR/TE) 200/23 ms, δ/Δ 2.5/12 ms. A zero-interpolation filling factor of 10% was used across the phase and slice encoding directions. One brain was imaged at a higher resolution of 0.075 mm in the anteroposterior dimension, and was resliced to 0.10 mm isotropic resolution prior to subsequent analysis. For each dataset, two *b*_0_ (b-value = 0 s mm^−2^) images and 30 diffusion-weighted (b-value = 5000 s mm^−2^) volumes were acquired. The 30 diffusion-gradient directions used were evenly distributed over a hemisphere using the electrostatic repulsion method. No image averaging was used. Total acquisition time was approximately 14.5 h per brain. In addition to dMRI, each brain was also imaged with the FLASH imaging protocol to obtain structural images with the following parameters: MTX 654 × 380 × 280, FOV 19.6 × 11.4 × 8.4 mm (0.03 mm isotropic voxels), TR/TE 50/12 ms, flip angle of 30°. No image averaging was used. Acquisition time was 0.7 h per brain.

Data processing was performed on a high-performance computing cluster with 1600 CPU cores and 8 TB of RAM housed at the Queensland Brain Institute. Following image reconstruction and orientation, *b*_0_ volumes were averaged and used to generate a binary brain mask. Scalar diffusion metric images were generated via weighted least-squares diffusion tensor estimation, using DTIFIT from the FSL software package (Behrens et al., 2003) (FSL v 5.0; https://fsl.fmrib.ox.ac.uk/fsl/fslwiki). Color fractional anisotropy (FA) maps were generated by the product of primary eigenvector of the diffusion tensor and FA. Fiber orientation distribution (FOD) was estimated with MRtrix (v0.2.7; www.nitrc.org/projects/mrtrix) by constrained spherical deconvolution (CSD) using a similar procedure to those previously described (Liu et al., 2016). Briefly, a mask of high anisotropy voxels was obtained by eroding a binary mask of a fractional anisotropy map; masked voxels were subsequently thresholded at an intensity of 0.7. Voxels that survived erosion and thresholding were assumed to represent a single fiber mask, which was subsequently used to estimate the response function spherical harmonic coefficients with a maximum harmonic order (l_max_) = 6.

### 2.3. Reference atlas construction and registration

**Fig. 1.**
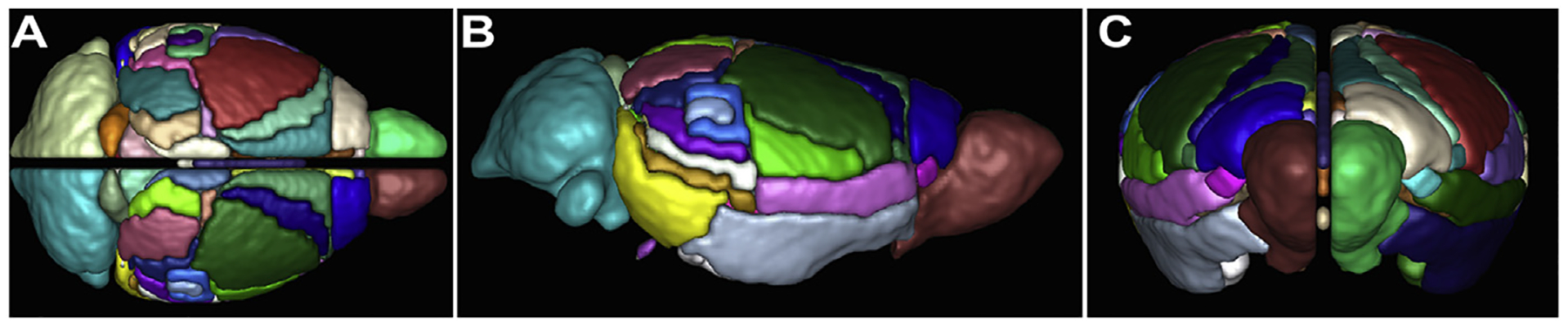
3D representation of the adult mouse brain atlas. Each colour indicates a distinct ROI in the atlas, corresponding to the areas outlined in Table 1, in horizontal (A), sagittal (B) and coronal (C) views.

Segmentation was performed by combining two previously established *ex vivo* MRI-based adult mouse brain atlases: a cortical atlas from the Centre for Advanced Imaging at The University of Queensland (CAI atlas) (Ullmann et al., 2013), and a subcortical atlas created by the lab of Prof. Susumu Mori of Johns Hopkins University (JHU atlas) (Chuang et al., 2011). The methods used to combine the two atlases have been described previously (Liu et al., 2016). Briefly, the JHU atlas was spatially transformed to the CAI template with affine transformation followed by nonlinear deformation by Advanced Normalisation Tools (ANTs; http://stnava.github.io/ANTs/). Small, functionally related areas were combined for a total of 76 regions (38 regions per cerebral hemisphere); the components of each region are summarized in Table 1 and shown in Fig. 1). To minimize areal overlap due to differences in brain registration and partial volume effects at dMRI resolution, the perimeter of each cortical area was eroded by two voxels. Preliminary analysis of connectivity in partial CCD and complete CCD brains identified spurious interhemispheric streamlines that skipped across the longitudinal fissure. To prevent this from affecting the final estimation of connectivity, a five voxel-wide (0.5 mm) exclusion region of interest (ROI) was manually drawn at the telencephalic midline between the hemispheres.

A virtual callosotomy was also conducted for comparative purposes, the rationale for which has been previously discussed in-depth by Owen and colleagues (Owen et al., 2013a,b). In brief, virtual callosotomies introduce an additional series of controls lacking callosal connections, which can be compared with CCD brains, and allows for the differentiation of network differences in CCD brains attributable to absence of callosal fibers as opposed to novel connectivity outside of the CC. This assists in attributing differences in connectivity and network characteristics in CCD, compared to normal CC controls, to the reorganization of connections rather than just the absence of callosal connectivity. The virtual callosotomies were performed by manually drawing a midsagittal exclusion ROI over the CC in the native diffusion space of normal CC brains. The averaged *b*_0_ volume of the dMRI volume of each brain was mapped to the 0.1 mm isotropic adult mouse brain model by affine and diffeomorphic transformation using the “greedy symmetric normalisation model” in ANTs; symmetric cross-correlation was used as a similarity metric with a maximum of 60 × 180 × 40 iterations (Avants et al., 2011). The inverse transformation was applied to the reference atlas to bring anatomical labels into the individual image space of each brain.

### 2.4. Probabilistic tractography and connectome construction

CSD-based probabilistic tractography (using the SD PROB algorithm) was performed using in-house scripts based on MRtrix (v0.2.7) (Tournier et al., 2012) using procedures similar to those described previously (Moldrich et al., 2010; Liu et al., 2016). The tractography tracking parameters were as follows: FOD cut-off = 0.1, maximum number of 10,000 streamlines, maximum number of attempts = 1,000,000, step size = 10 μm, curvature threshold = 11.5°/step, minimum/maximum streamline length = 0.5/20 mm. Probabilistic tractography was initiated from each cortical and subcortical anatomic label. Whole brain connectivity matrices were generated using a two-step approach; first, streamlines were seeded from each atlas-based region and the fraction of the total number of streamlines passing through each voxel relative to the total number generated was used to create a connectivity map for each seed region (76 tractography results). The connectivity map of each seed was masked by each of the remaining 75 anatomic labels; a putative connection between a pair of regions was retained for further analysis when the average of the ratio between the sum of connected voxel values and the volume of the region was greater than zero. Next, tractography between each pair of potentially connected regions identified in the first step was performed whereby both anatomic labels were specified as “seed” and “include” regions for streamline generation. The same threshold was applied to the resulting tractography results and the surviving connections were entered into a 76 × 76 connectivity matrix for each brain. For subsequent analysis of connectomes, connections were further weighted by the mean FA across voxels assigned a connectivity value greater than 0.001 for each area pair combination; consequently, the connection ‘strength’ between cortical regions was also weighted by the tissue microstructural features of the neural tract connecting them generated by tractography, rather than within the atlas-defined area.

### 2.5. Connectome visualization

Consensus connectomes were constructed for each condition: complete CCD, partial CCD, normal CC and virtual callosotomy controls. Connections present in half of the individual connectomes of each group were included in the consensus connectome and connection strength (based on FA) was averaged across all brains. Connectomes were visualized in anatomical layouts or circular layouts constructed using the BrainNet Viewer visualization tool (https://www.nitrc.org/projects/bnv/) (Xia et al., 2013) and Circos software (https://www.cpan.org/ports; Krzywinski et al., 2009), respectively. For anatomical layouts, nodes were plotted at the center of gravity for each atlas region (calculated using the center of gravity function in FSL) and colored according to degree using a custom colormap. Edges between nodes were weighted according to mean FA across the tract. A complete description of the methods used to generate circular connectomes, also known as connectograms, is outlined by Irimia et al. (2012). Briefly, atlas regions were represented as uniquely colored radially oriented segments, ordered according to lobe assignment, and separated into left and right hemispheres. Within each lobe, parcellations were arranged approximately according to the order of their location along the dorso-ventral axis. Individual node measures were calculated by methods described in the following section, averaged across all individual brains for each condition, and displayed in concentric radially oriented segments from outside in: node strength, local efficiency, betweenness, clustering, and module assignment. Edges are weighted according to the streamline count for each pairwise area connection and are colored according to mean FA across the tract (red = top tertile, green = middle tertile, blue = bottom tertile).

### 2.6. Network and node-based analysis

Comparisons of graph theory measures at the network and node level of structural connectomes were performed using GRETNA (Wang et al., 2015) (https://www.nitrc.org/projects/gretna/). All networks were proportionally thresholded based on sparsity in GRETNA (Wang et al., 2015), using thresholds ranging from 0.05 to 0.13 (incrementing in steps of 0.01), to exclude weak or spurious connections while simultaneously maintaining the same network density across all tested conditions. Mean nodal strengths, mean nodal betweenness, network global efficiency, mean nodal local efficiency and mean nodal clustering coefficient were calculated for each connectome for each threshold level, as well as their corresponding area-under-the-curve (AUC) across the full threshold range.

The strength of a node was defined as the sum of the weights of the edges directly connecting that node to another, ‘neighboring’, node; the strengths of all nodes in a connectome were averaged to obtain the mean weighted degree of the network. The betweenness of a node was defined as the percentage of all shortest paths between each pair of nodes in a network that contains that node (Brandes, 2001). Mean betweenness was calculated by averaging the betweenness of all nodes in the network. Global efficiency was defined as the inverse of the mean path length between all nodes in the network. Local efficiency was defined analogously, but for local neighborhoods of nodes. The clustering coefficient was defined for each node as the ratio of neighboring nodes that are also nodes of each other (Watts and Strogatz, 1998). For all measures, calculations were made on weighted edge measures based on the average FA value as described in the preceding section, and the AUC for all thresholds was calculated.

The modularity of each network was determined by the brain connectivity toolbox implementation of the Louvain community detection algorithm (Rubinov and Sporns, 2011). Gamma was optimized for modularity for normal CC controls (γ = 1.2), which was applied to other callosal conditions. The participation coefficient was calculated by utilizing the previously determined consensus community partitions, as described previously (Guimerà and Amaral, 2005), and were displayed using the Gephi toolkit (Bastian et al., 2009).

### 2.7. Statistics

All statistical comparisons were performed using the GraphPad Prism software package (v 7.0a for Mac, GraphPad Software, La Jolla California USA). Statistical comparisons between the AUC of all measures were performed using unpaired two-tailed Student’s t-tests with Holm-Sidak correction for multiple comparisons and a threshold for significance of p < 0.05. Normality of distribution of indicated datasets was assessed via D’Agostino-Pearson omnibus normality tests and differences in variance of datasets was probed with F tests (significance cut-off p < 0.05). To test if individual networks were more or less similar to other individual networks in both complete and partial CCD compared to controls, the average edge-wise correlation coefficient between each pair of brains within each callosal condition was calculated. The nine averaged values obtained for each network (i.e. ten comparisons excluding the correlation of a network with itself) were averaged to obtain a measure of similarity of each individual network to other individual networks with the same callosal condition. These values were calculated across the whole network, and for interhemispheric and intrahemispheric connections separately, to determine the relative contribution of both to overall variability.

To identify subnetworks of connections that differed significantly between callosal conditions, the NBS toolbox was utilized (Zalesky et al., 2010). The NBS controls for the family-wise error rate (FWER), which would normally complicate comparisons of every connection in a network, by performing permutation testing on connected graph components comprised of a set of connections that reach an initial test-statistic threshold. Graph components that reached p < 0.05 (FWER-corrected) were considered to be significantly different between conditions (number of permutations = 5000, tested across a range of integer thresholds between 2 and 5). Due to the variability in CCD connectomes, an additional discovery strategy was employed to identify novel and preserved individual connections in partial CCD. In this case, novel connections were defined as network edges that were present in two or more brains per condition (i.e. 20% penetrance), but did not exist in any normal CC brain.

### 2.8. ROI-based tractography analysis

Tractography and functional imaging studies in CCD humans have suggested that alternative commissures in the midbrain and forebrain may compensate for the absence of callosal fibers (Tovar-Moll et al. 2007, 2014; Tyszka et al., 2011, Lazarev et al., 2016). To determine the specific connectivity of alternative commissures in BTBR N2 mice we performed ROI-based tractography of the anterior commissure, posterior commissure, hippocampal commissure and CC. To determine the connectivity of these tracts, ROIs were drawn bilaterally in two parasagittal planes, approximately 0.3 mm to the left and right of the midsagittal plane, in the diffusion image space of each individual brain. These ROIs were then used to generate streamlines for each commissure using identical parameters to those used to generate structural connectomes, with 50,000 streamlines selected for each tract before tracking was terminated. Individual tractography results were converted to connectivity maps by calculating the fraction of streamlines passing through each voxel as a proportion of the total number of streamlines generated. The resulting connectivity maps were each warped to the common atlas space using warps generated in the process of atlas registration, and were then averaged across the 10 animals in each condition to produce a single mean streamline density map of each condition. To reconstruct hetero-topic tracts, tractography was seeded from the frontal association cortex. Inclusion ROIs were specified as bilateral parasagittal callosal ROIs, and the ipsilateral frontal and contralateral occipital projections of the CC. Exclusion ROIs were specified as the midline exclusion ROI specified by the brain atlas, as well as contralateral frontal and ipsilateral occipital projections. CSD-based probabilistic tractography was performed with the following parameters: FOD cut-off = 0.2, maximum number of 10,000 streamlines, maximum number of attempts = 1,000,000, step size = 10 μm, curvature threshold = 11.5°/step, minimum/maximum streamline length = 5/40 mm.

### 2.9. In utero electroporation and tissue collection

*In utero* electroporation of embryos and postnatal tissue collection for histological validation of ectopic tracts was performed as described previously (Kozulin et al., 2016; Suárez et al., 2014; Fenlon et al., 2017). In brief, F1 females were placed in the same cage as BTBR males overnight and where a vaginal plug was detected on the next day, this was considered embryonic day (E)0. For electroporation surgery, pregnant dams were anesthetized with an intraperitoneal injection of ketamine and xylazine (120 mg/kg ketamine; 10 mg/kg xylazine) at E15, when *in utero* electroporation is well established to predominantly label layer 2/3 neurons of the cortex (Suárez et al., 2014). Dams underwent a laparotomy to expose the embryos, and each embryo was injected with 0.5–1 μL of pCAG-tdtomato plasmid (1 μg/μL concentration, Clontech tdTomato fluorophore subcloned into pCAG) in the right lateral ventricle using a pulled glass pipette and a Picospritzer II (Parker Hannifin). 3 mm paddle electrodes (Nepagene) connected to an ECM 830 electroporator (BTX Harvard Apparatus) were then placed over the presumptive right occipito-temporal cortex, and five 100 ms square wave pulses at 35 V were delivered to transfect the plasmid into the underlying developing neurons. After each embryo had been electroporated, the uterine horns were replaced into the abdominal cavity which was sutured closed. The dam recovered on a warmed pad and was provided self-administered buprenorphine (0.2 mL of a 0.026 mg/mL solution) injected into a MediGel (ClearH2O) for pain relief. Dams gave birth to live pups at E19/P0 and the pups were collected between P15-P20. Pups were anaesthetised via intraperitoneal injection of 0.2–0.4 mL of sodium pentobarbitone (1/50 v/v Lethabarb^™^, Virbac) and then transcardially perfused with saline followed by 4% PFA. Brains were removed and immersed in 4% PFA for post-fixation for at least 48 h.

### 2.10. Histological tissue processing

Brains were dissected from the skull and screened under a fluorescence microscope for the presence and consistency of size and location of the fluorescence area indicating the region of transfection. Any brains that did not have comparable parameters of transfected cell fields were excluded from further processing. Brains were embedded in 3.5% noble agar and sectioned on a vibratome horizontally at 50 μm. Immunohistochemical staining was performed on mounted sections against tdTomato (to enhance fluorescence) using goat-anti-tdTomato primary antibody (Sicgen 8181–200; 1:1000) with Alexa Fluor 555 donkey-anti-goat secondary antibody (Invitrogen; 1:500) and counterstained with DAPI (Invitrogen; 1:1000).

### 2.11. Histological image acquisition and data analysis

At least 10 sections throughout the dorsolateral extent of each brain were carefully examined and each animal categorized as control, partial or complete CCD using the criteria of: 1. the presence or absence of any callosal fibers; 2. the presence or absence of complete or partial Probst bundles and; 3. the size of the callosal tract at the midline. Any brains that were considered a borderline case for any of these three criterion were excluded from further analysis. Ten animals that clearly satisfied the control criteria and ten that satisfied the partial CCD criteria that had comparable size and location of fluorescent transfected fields and were age-matched were selected for further analysis. Sections were matched in the dorso-ventral position across individual brains prior to analysis. High resolution confocal images were acquired of the contralateral frontal cortex (ROI 1 and 2) and contralateral homotopic cortex (ROI 2) using a Diskovery spinning disk confocal microscope (Spectral Applied Research) with two cCMOS cameras (Andor Zyla 4.2) and a 20x/0.75 NA air objective (Nikon) controlled with Nikon NIS software. Widefield images of entire sections were acquired with a Zeiss upright Axio-Imager Z1 microscope fitted with Axio-Cam HRm camera and captured with Zen software.

### 2.12. Analysis of histological images

To analyze the degree of labeled axonal presence in select brain regions, 350 μm × 350 μm ROIs were placed over the center of each of these regions, and the fluorescence intensity was calculated within each square with Fiji (Image J). These values were normalized against the fluorescence of an ROI of the same size placed on a “background” section of the same brain that did not contain any signal, in order to account for differences in background fluorescence, as described previously (Fenlon et al., 2017). Normalized fluorescence intensity values for each ROI were first tested for assumptions of normality of distribution using a D’Agostino & Pearson Normality Test and then each ROI was statistically compared for control versus partial CCD animals using unpaired two-tailed Student’s t-tests in the case of normally distributed data, or a Mann-Whitney U test in the case of data that did not meet the assumption of normal distribution (Graphpad Prism software package).

## 3. Results

### 3.1. The BTBR N2 mouse model displays variable CCD phenotypes

**Fig. 2.**
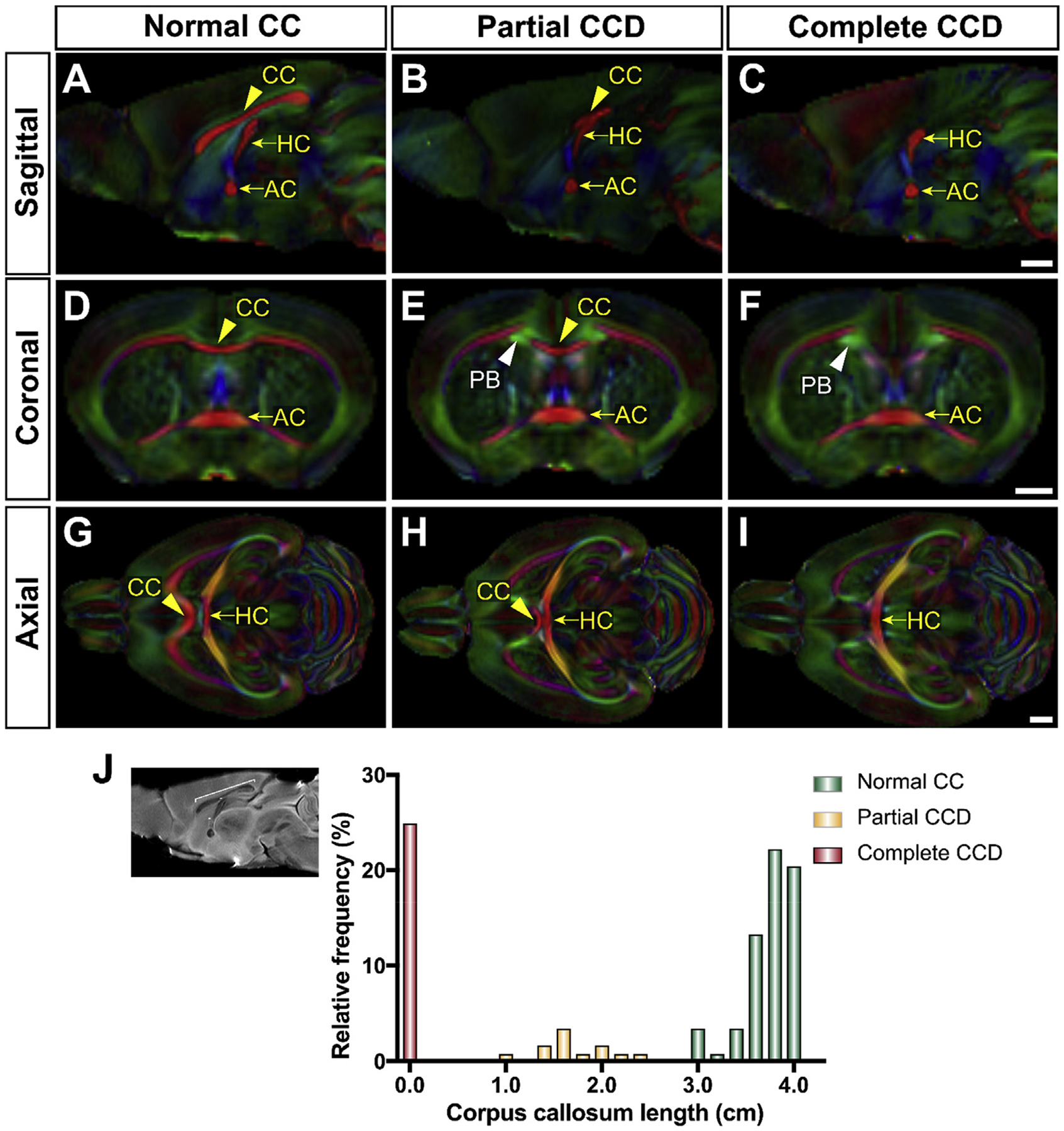
Color fractional anisotropy maps of representative of the distribution of callosal phenotypes in BTBR N2 littermates. BTBR N2 mice (n = 112) were classified according to the anteroposterior length or absence of the CC as normal CC (A,D,G), partial CCD (B,E,H) or complete CCD (C,F,I). Compared to normal CC (yellow arrowheads) mice, partial CCD mice displayed reduced anteroposterior length of the corpus callosum (CC, yellow arrowhead in B) and Probst bundles (PB, white arrowhead in E), but intact hippocampal commissure (HC) and anterior commissure (AC; yellow arrows). Complete CCD mice displayed complete absence of callosal fibers (C), an intact hippocampal commissure and Probst bundles (white arrowhead in F). Relative frequency distribution of CC lengths clearly demonstrates the three distinct subsets of callosal phenotypes in BTBR N2 mice (J). A-I are color coded fractional anisotropy maps where red color denotes medial-lateral projecting fibers, green denotes anterior-posterior projecting fibers, and blue denotes superior-inferior projecting fibers. HC = hippocampal commissure. AC = anterior commissure. PB = Probst bundle. Scale bars = 1 mm.

The F2 BTBR x C57Bl/6J intercross has been demonstrated to result in progeny with a range of corpus callosum sizes (Jones-Davis et al., 2013). We anticipated that the BTBR N2 cross, being enriched for potentially causative BTBR alleles for CCD, would display a corresponding increase in the proportion of mice with complete or partial CCD. To determine whether this was the case, BTBR N2 mice were first classified into normal CC, partial CCD and complete CCD groups based on the antero-posterior length of the CC in single direction dMRI images taken at in the midsagittal plane (distribution of corpus callosum lengths in Fig. 2J). 28 (25%) animals demonstrated complete CCD (red column in Fig. 2J), and 69 (62%) animals demonstrated a normal corpus callosum (defined as an anteroposterior length greater than 3 mm based on previously published corpus callosum lengths in wild type C57BL/6J mice (Jones-Davis et al., 2013); green columns in Fig. 2J). Corpus callosum length of 15 (13%) animals fell into a distinct distribution between these two peaks, with callosal lengths between 1 mm and 2.5 mm; these mice were hereafter classified as partial CCD (yellow columns in Fig. 2J).

**Fig. 3.**
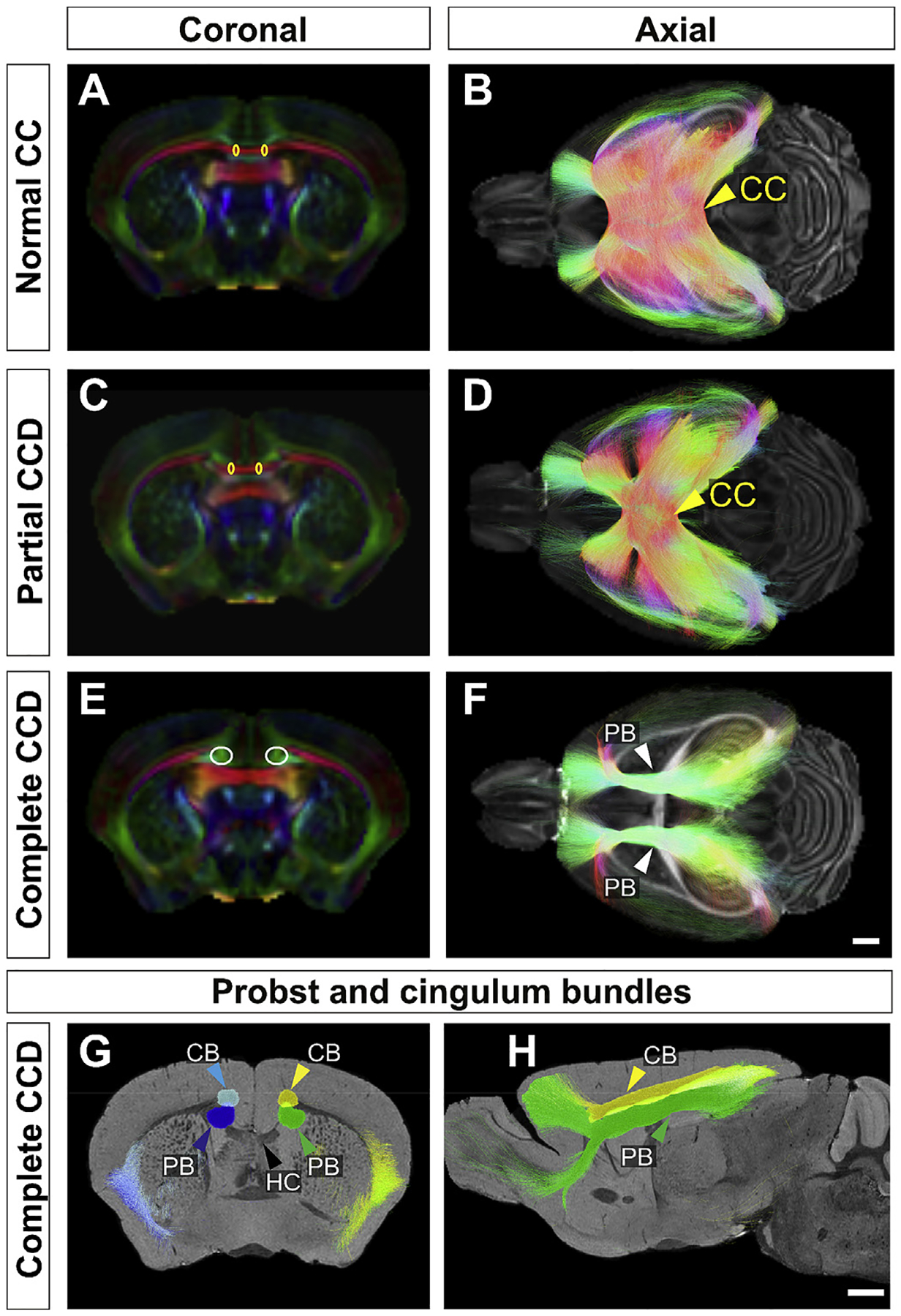
Tractography of the normal corpus callosum, the callosal remnant in partial CCD, and longitudinal bundles of Probst in CCD BTBR N2 mice. Bilateral ROIs (ellipses in A,C,E) were drawn over color FA maps (A,C,E) to reconstruct the CC in control (A, B) and partial CCD (C,D), and longitudinal bundle of Probst (PB) in complete CCD (E,F). All streamlines passing through the CC are shown for normal CC (B) and partial CCD (D) mice, projected over FA maps. In complete CCD, callosal axons are reorganized into Probst bundles projecting along the anteroposterior axis (PB; F). These bundles (dark blue and green in G and H) can be differentiated by CSD tractography from the adjacent cingulum bundle (CG; light blue and yellow in G and H). HC = hippocampal commissure, black arrow; scale bars = 1 mm; scale bar in H for panels A, C, E, G and H; scale bar in F for B, D and F.

To delineate the anatomical relationships between commissures and associated tracts in the heterogeneous callosal conditions of BTBR N2 mice, color FA maps were generated (Fig. 2A – I). This confirmed normal gross callosal morphology of normal CC mice (Fig. 2A, D and G), a callosal remnant and antero-posteriorly-oriented smaller Probst bundle tracts in partial CCD mice (Fig. 2B, E and H), and the presence of large Probst bundles and complete absence of crossing fibers at the midline in complete CCD mice (Fig. 2C, F and I). In normal CC mice, the CC could be reconstructed as a band of mediolateral streamlines (Fig. 3A and B). The CC could still be reconstructed in the partial CCD mice, though it contained fewer streamlines and there were deficits in some callosal projections (Fig. 3C and D). In CCD, aberrant fibers forming the Probst bundles were reconstructed by tractography as longitudinally-oriented intra-telencephalic projections that do not cross the midline (Fig. 3E and F) and run adjacent to, but distinct from, the cingulum bundle (Fig. 3G and H). In CCD mice, the Probst bundle consistently projected between the frontal pole and the ipsilateral posterior cortex, closely resembling previous descriptions of the Probst bundle in acallosal mice (Ren et al., 2007) and humans (Utsunomiya et al., 2006; Tovar-Moll et al., 2007).

### 3.2. Reorganization of the structural connectome in mice with complete and partial CCD

**Fig. 4.**
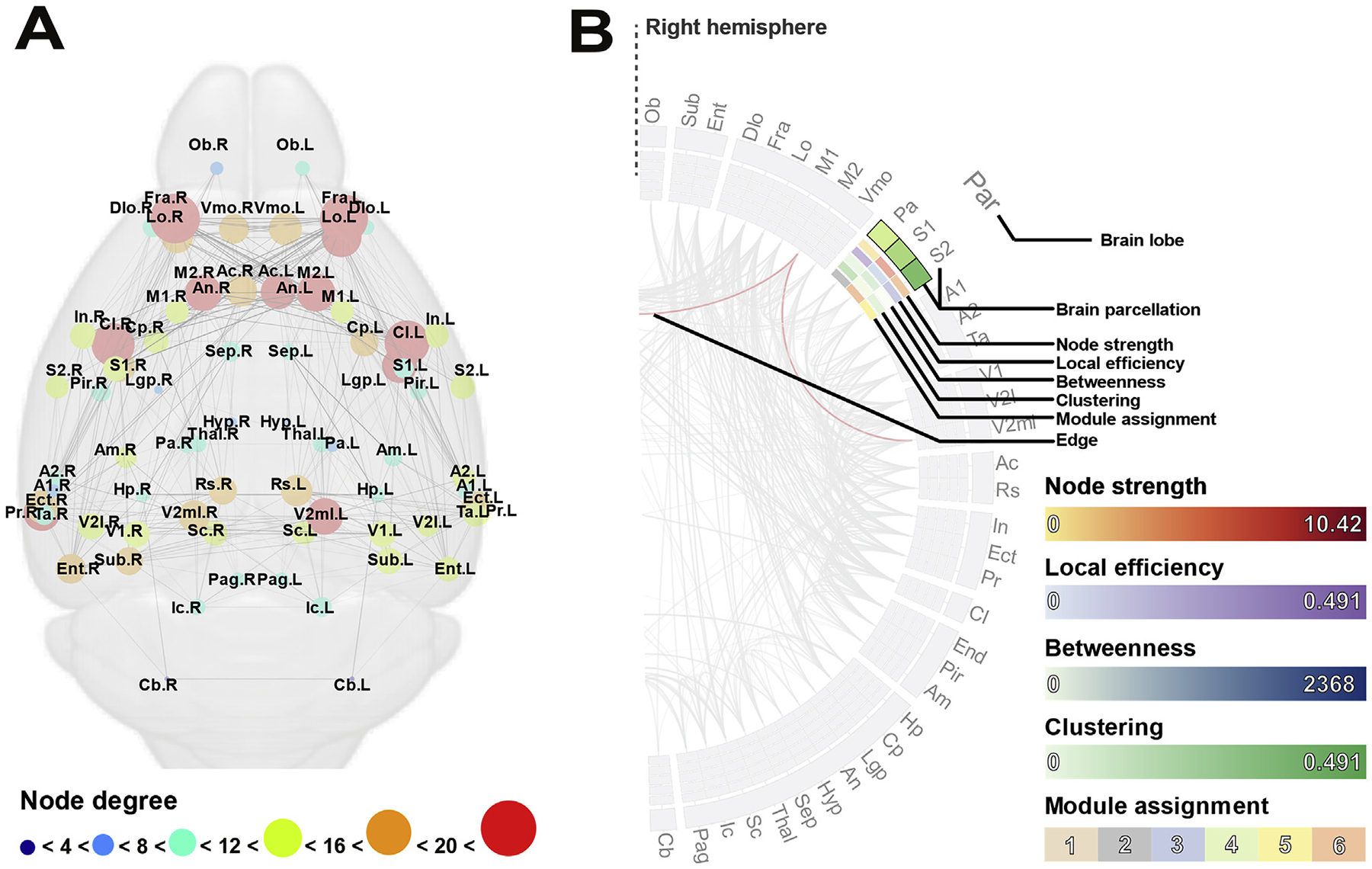
Example structural connectomes in anatomical (A) and circular (B) representations. In axial connectomes (A) cortical areas are plotted in anatomical layout corresponding to the center of gravity of each ROI in the adult mouse brain atlas. Cortical areas are represented as circles scaled and colored according to consensus degree (see legend below A); edges between nodes represent structural connections generated by tractography and are weighted according to average strength as determined by mean tract FA. (B) An example of one half of a circular connectome, corresponding to one hemisphere, highlighting the key features for the parietal lobe. Brain areas (labeled in the outer circle), which are grouped into lobes (labeled outside of brain areas), are arranged according to anatomical location. Anterior regions are plotted at the top of the circle, and posterior and subcortical regions are plotted at the bottom of the circle. Nodal properties are plotted in the inner circles from outside in: node strength, local efficiency, betweenness, clustering and module assignment. Indicative color maps for each measure are shown in the adjacent legend. Edges between regions in the circular connectome (two example edges are colored in red) are weighted according to streamline count and are colored according to mean tract FA.

**Fig. 5.**
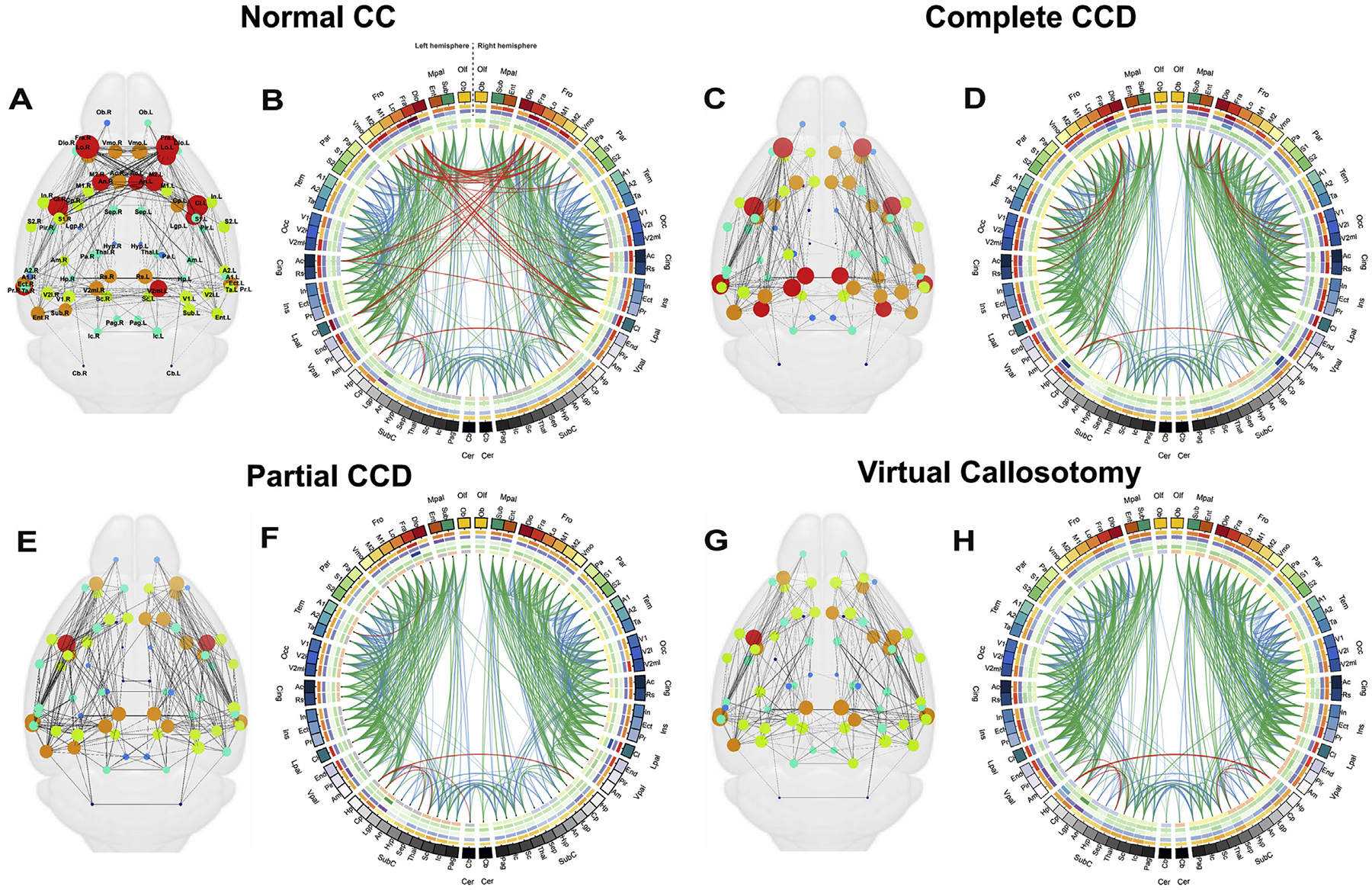
Consensus structural connectomes for BTBR N2 callosal phenotypes demonstrate reorganization of structural connectivity in complete and partial CCD mice. Consensus structural connectomes are displayed in axial (A,C,E,G) and circular (B,D,F,H) orientations for normal CC (A,B), complete CCD (C,D), partial CCD (E,F) and virtual callosotomy controls (G,H). Connections that comprise each connectome were present in at least half of all animals for each condition. Node characteristics and edge weights were calculated by averaging across all individual connectomes. The abbreviations and components of each brain region are listed in Table 1. See Fig. 4 for explanation and legends.

To compare whole-brain structural connectivity between complete CCD, partial CCD mice and normal CC BTBR N2 littermates, consensus structural connectomes were constructed for each condition. A virtual callosotomy was performed on normal CC brain scans by drawing an exclusion ROI over the midsagittal CC. The rationale behind the virtual callosotomy was to produce an additional series of control connectomes without callosal fibers, but also lacking any reorganization of connections seen in CCD brains (Owen et al., 2013a,b). Differences between CCD connectomes and either normal CC or virtual callosotomy connectomes can therefore be interpreted in the context of structural brain reorganization rather than due solely to the absence of callosal fibers. An example of the layout of the connectomes used is displayed in Fig. 4. Consensus axially-oriented (Fig. 5A, C, E and G) and circular (Fig. 5B, D, F and H) connectomes were constructed for normal CC (Fig. 5A and B), complete CCD (Fig. 5C and D), partial CCD (Fig. 5E and F) and virtual callosotomy conditions (Fig. 5G and H). Compared to the normal CC consensus connectome, complete CCD and partial CCD connectomes demonstrate reorganization of edges between network nodes, with a notable reduction in interhemispheric connections between cortical regions, but preservation of connections between hippocampus, cerebellum and subcortical structures. The complete CCD connectome displays highly weighted edges between ipsilateral frontal, parietal, temporal and occipital regions, and a similar degree of connectivity of high-degree nodes (Fig. 5C and D) compared to normal CC controls (Fig. 5A and B). The consensus partial CCD connectome (Fig. 5E and F) displays some interhemispheric edges, with heterotopic edges between areas within the left frontal lobe and cingulate with contralateral ventral pallial, lateral pallial and subcortical structures.

**Fig. 6.**
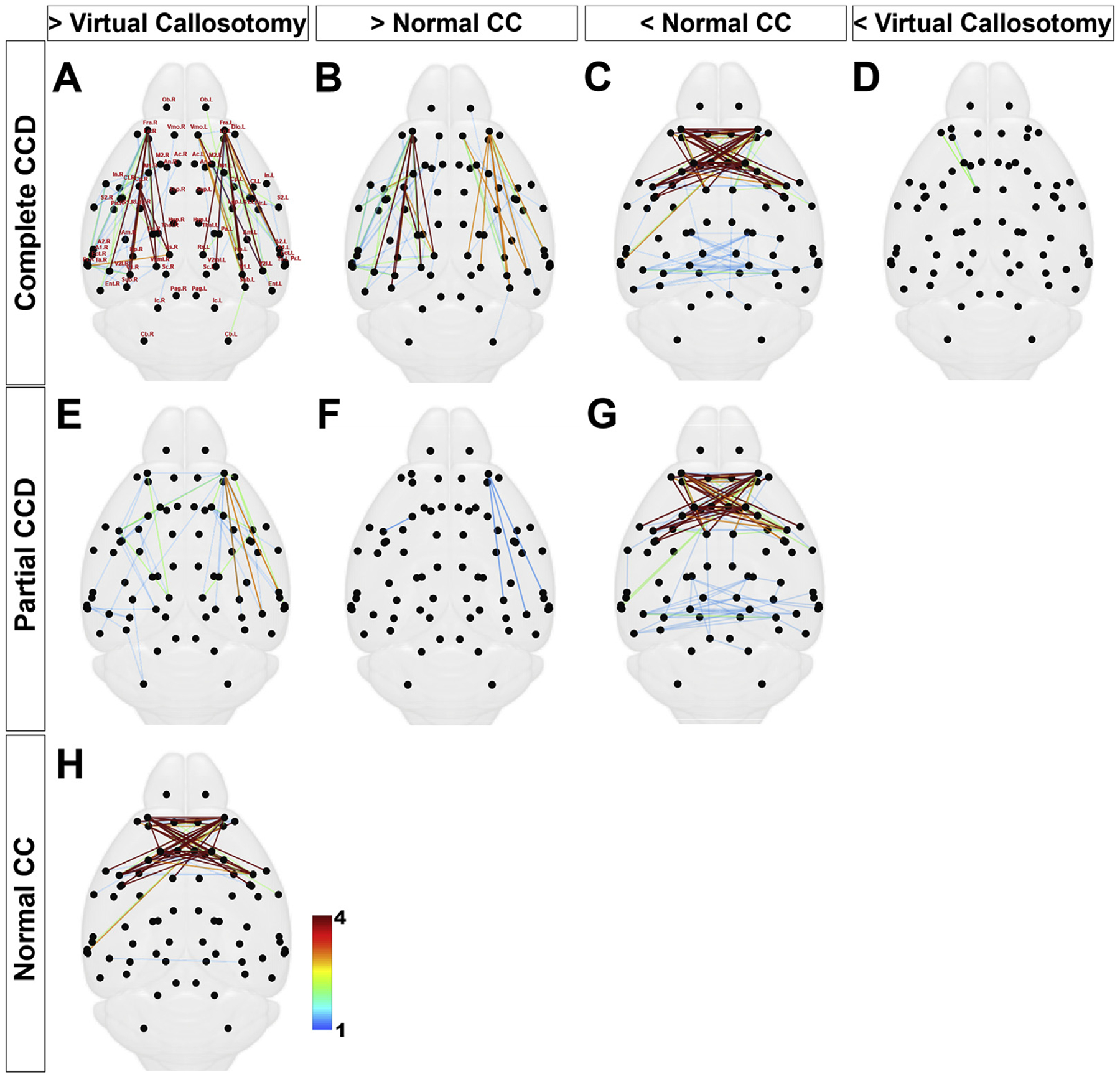
NBS results for comparisons between structural connectomes for BTBR N2 callosal conditions. NBS results for whole brain connectomes of complete CCD (A-D), partial CCD (E-G) and normal CC (H) compared to normal CC and virtual callosotomy controls (contrasts indicated by headings of columns). For example, A displays edges in complete CCD (row title) that are significantly greater than virtual callosotomy controls (column title). Edge color corresponds to the number of thresholds that were considered significant for a given comparison, from 1 to 4 (for thresholds tested from 2 to 5 inclusive). Non-significant results (for instance, normal CC < virtual callosotomy) are not shown.

Differences between consensus connectomes are further demonstrated in Fig. 6, which shows differences detected by NBS between complete (Fig. 6A – D) and partial CCD (Fig. 6E – G), and normal and virtual callosotomy controls. Edge color in each connectome corresponds to the number of times an edge was defined as significantly different from the control condition for a range of test statistics (integers with a range 2–5). Two major subnetworks were identified with increased connectivity in complete CCD compared to both virtual callosotomy and normal CC conditions (edges in Fig. 6A and B). These comprised long-distance connections between predominantly prefrontal regions to ipsilateral posterior cortical regions including visual and auditory regions. Two interhemispheric subnetworks were identified with decreased connectivity in complete and partial CCD compared to normal CC controls (edges in Fig. 6C and G respectively), comprising bilateral frontal, parietal and occipital regions, as well as the septum and claustrum. Partial CCD brains demonstrated a similar increase in intrahemispheric subnetworks compared to complete CCD when compared to the virtual callosotomy controls, albeit across a reduced range of thresholds (Fig. 6E). Compared to normal CC controls, only intrahemispheric connections involving the left frontal association cortex were significantly increased in partial CCD, whereas a connection between motor cortex and claustrum was increased in the contralateral hemisphere (Fig. 6F). No connections were identified in partial CCD that were reduced in size compared to virtual callosotomy controls, whereas a small subnetwork comprising the right septum and frontal regions was significantly reduced in complete CCD (Fig. 6D). We observed similar interhemispheric subnetworks that were greater in normal CC compared to the virtual callosotomy controls (Fig. 6H as complete (Fig. 6C) and partial CCD (Fig. 6G). While the posterior interhemispheric subnetwork was reduced in extent to a single edge in the comparison between normal CC and virtual callosotomy, this subnetwork was only identified at a single threshold across all comparisons. Importantly, we did not identify any significantly reduced intrahemispheric subnetworks in normal CC compared to virtual callosotomy, suggesting that virtual callosotomy is an appropriate control for considering the absence of callosal connections without compensatory reorganization.

To examine how differences in edge distribution may influence specific brain areas, the AUC for node properties across a range of network thresholds were compared between callosal conditions using unpaired two-tailed Student’s t-tests (Holm Sidak correction for multiple comparisons, alpha <0.05). Complete and partial CCD connectomes demonstrated multiple significant differences in node-specific measures compared to virtual callosotomy controls, which could be interpreted as the consequences of structural reorganization that occurs when the CC fails to form correctly. The nodes that demonstrated a significant increase or decrease in measures relative to controls are outlined in Table 2. Complete CCD mice had significant increases in weighted degree of bilateral frontal regions, and left visual and hippocampal regions. Partial CCD mice demonstrated a unilateral increase in nodal degree of the left frontal association area, which is consistent with the presence of one-sided interhemispheric connections that involve this area in the consensus connectome.

### 3.3. Differences in global network measures between brains with distinct callosal phenotypes

**Fig. 7.**
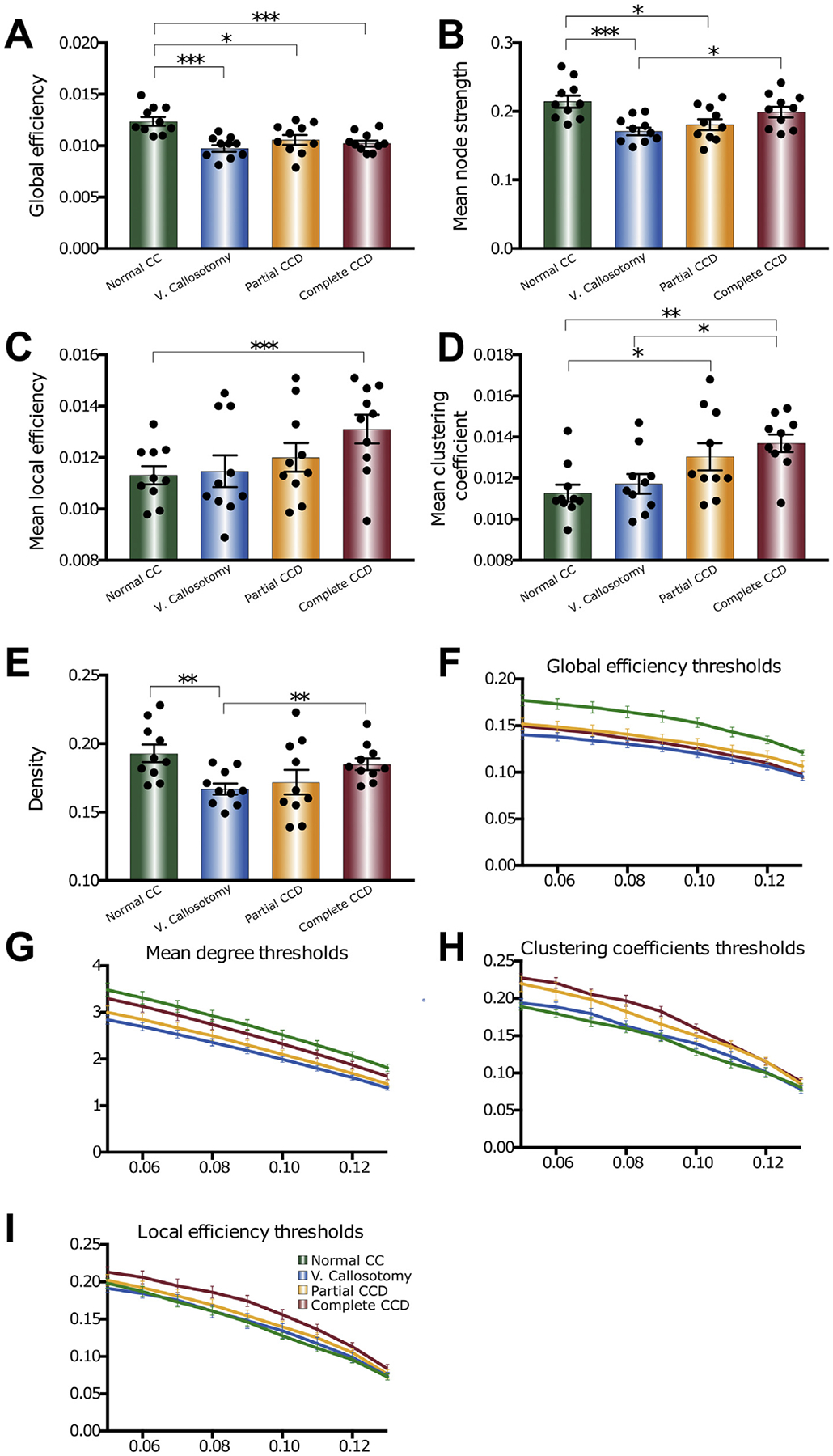
Whole brain network measures in BTBR N2 callosal phenotypes and virtual callosotomy controls demonstrate reorganization of the CCD connectome. Network measures were calculated for a range of network sparsity, and comparisons were performed on the AUC of each measure (A-E) across a range of network thresholds (F-I). Global efficiency (A and F), mean degree (B and G), mean local efficiency (C and H), mean clustering coefficient (D and I) and overall network density (E) were compared for complete CCD, partial CCD, normal CC and virtual callosotomy controls. *p < 0.05, **p < 0.01; ***p < 0.001 by either unpaired two-tailed Student’s t-tests (for datasets that were normally distributed, with a Welch’s correction applied where variance was significantly different) or Mann-Whitney U tests (for datasets that were not normally distributed). Data points represent network measures of individual brains, and values are represented as mean ±SEM.

The structural connectome of humans with CCD is shifted towards reduced global efficiency and increased local efficiency, with associated increases in mean normalized betweenness and mean clustering coefficient, but preserved network mean degree (Owen et al., 2013a,b; Jakab et al., 2015). To test whether CCD mice demonstrate comparable differences in global network organization, AUC weighted network summary metrics were calculated for whole brain global efficiency, degree, mean local efficiency, and mean clustering coefficient for each individual mouse (Fig. 7). Global efficiency was significantly reduced in the virtual callosotomy (p = 0.0001), partial CCD (p = 0.0108) and complete CCD (p = 0.0005) groups compared to the normal CC controls (Fig. 7A). Node strength (Fig. 7B) was not significantly different between complete CCD and normal CC controls (p = 0.21). Mean node strength was significantly reduced, however, in partial CCD and virtual callosotomy compared to normal CC control (p = 0.01 and p = 0.0007, respectively), and in virtual callosotomy compared to complete CCD (p = 0.01). Weighted mean local efficiency (Fig. 7C) and mean clustering coefficient (Fig. 7D) demonstrated similar increases in complete CCD brains as compared to both normal CC controls (p = 0.0139 and p = 0.0031, respectively) and virtual callosotomy brains (p = 0.0637 and p = 0.0064 respectively), possibly reflecting a rearrangement of connectivity that favors local, intrahemispheric networks. Network density (Fig. 7E) was not significantly different between normal CC and either partial CCD (p = 0.0735) or complete CCD (p = 0.3246), though it was decreased in virtual callosotomy compared to complete CCD (p = 0.0066) and normal CC (p = 0.0031). In all measures, partial CCD brains followed similar trends to those displayed by complete CCD; however, these did not always reach significance. This may reflect partial CCD being an intermediate network phenotype between normal CC brains and complete CCD.

Together, these changes in overall network measures indicate that the structural connectome of the mouse CCD brain supports local connectivity and clustering at the expense of global efficiency, and that these changes are more extensive in complete CCD than partial CCD. Moreover, by comparing to virtual callosotomy networks, the reorganization of network edges in complete and partial CCD brains appears to compensate for the absence of callosal connections to partially rescue overall network structure.

### 3.4. Altered modular and hierarchical structure in complete and partial CCD mice suggest alternative hubs of structural connectivity

**Fig. 8.**
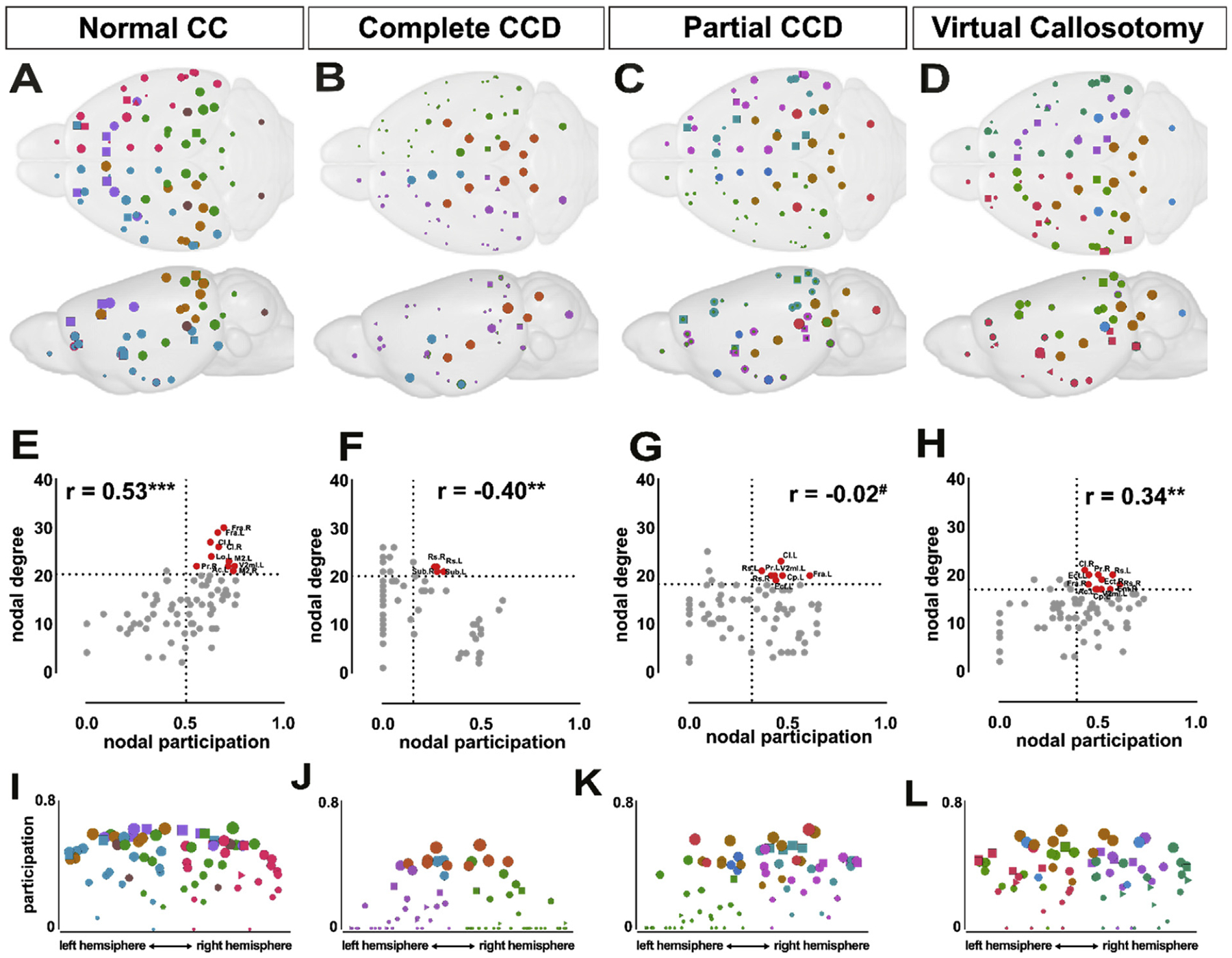
Altered modular and hub organization in complete and partial CCD mice compared to normal CC and virtual callosotomy controls. Modules were determined for the consensus connectome of each callosal condition by Louvain community detection (A,B,C,D). Connector hubs are denoted as squares, and provincial hubs as triangles. Colors denote community assignment; colors are arbitrary and do not correspond to the same community across conditions. Colors of nodes and edges denote module assignment of each brain area. XY scatter and linear regression of node degree and participation demonstrates a positive correlation coefficient (r) in normal CC controls (E) and virtual callosotomy controls (H), but inverse correlation in complete CCD (F), and no correlation in partial CCD (G). XY scatter of participation coefficient and mediolateral position of brain areas in normal CC (I), complete CCD (J), partial CCD (K) and virtual callosotomy (L). ***p < 0.0001, **p < 0.001, ^#^p = ns.

To further investigate how changes in structural connectivity lead to differences in network organization in CCD, the community organization and hub distribution in different callosal phenotypes was examined. A core organizational principle of the structural connectome is that nodes are segregated into highly intraconnected groups, termed ‘modules’, that have comparatively sparse outgoing connections to other modules (Rubinov and Sporns, 2010). The normal CC brain is organized into six modules: a bilateral frontal module, right and left inferolateral hemisphere modules, right and left-sided posterior modules comprising visual areas, and a module comprising cerebellum and hippocampus (Fig. 8A). In contrast, the complete CCD connectome is organized into four modules: left and right-sided modules comprising the majority of each cortical hemisphere, and two modules comprising basal ganglia and midbrain nodes, and subcortical nodes (Fig. 8B). The partial CCD brain is organized into six modules in a similar configuration to the complete CCD brain, but with asymmetrical division of the left hemisphere into medial and lateral divisions (Fig. 8C). The virtual callosotomy brain demonstrated a broadly similar organization of modules in the left and right hemispheres to normal CC control, though it lacked the bilateral frontal module (Fig. 8D).

Because complete and partial CCD structural networks demonstrate alterations in modular organization, we next investigated how these differences in community organization relate to the topological participation of individual regions. In the normal CC brain, participation positively correlated with nodal degree (Fig. 8E), consistent with previous results in the mouse brain (Rubinov et al., 2015). High participation, high nodal degree hubs in the normal CC brain include bilateral frontal association areas, bilateral claustrum, bilateral secondary motor cortices, right piriform, left anterior cingulate, left lateral orbital cortex and left secondary mediolateral visual cortex. In contrast, in complete CCD, there is an inverse correlation between nodal degree and participation (Fig. 8F); high participation-high degree nodes are the bilateral retrosplenial cortices and subiculum. Partial CCD showed a lack of positive correlation (Fig. 8G) with high participation-high degree nodes including left frontal association, ectorhinal and secondary mediolateral visual cortices, bilateral restrosplenial cortices, and left claustrum, piriform and caudate-putamen. Finally, virtual callosotomy controls demonstrate more overlap with normal CC, with positively correlated participation and degree (Fig. 8H), and a mixture of high participation-high degree nodes from both normal CC and CCD conditions including bilateral retrosplenial cortices, right frontal association, left anterior cingulate, piriform and entorhinal cortices and left caudate and putamen. In normal CC mice, high participation nodes (squares in Fig. 8I – L) were assigned to both medial and lateral cortical modules. In complete and partial CCD, high participation nodes were predominantly assigned to low degree medial subcortical regions (Fig. 8J and K). Together, these results suggest that CCD results in a reorganization of normal community structure, resulting in an inverse relationship between degree and participation between modules, and a shift in high participation nodes toward subcortical structures rather than cortical association areas.

### 3.5. Complete and partial CCD brains have a greater variability in interhemispheric and intrahemispheric connections compared to normal CC brains

**Fig. 9.**
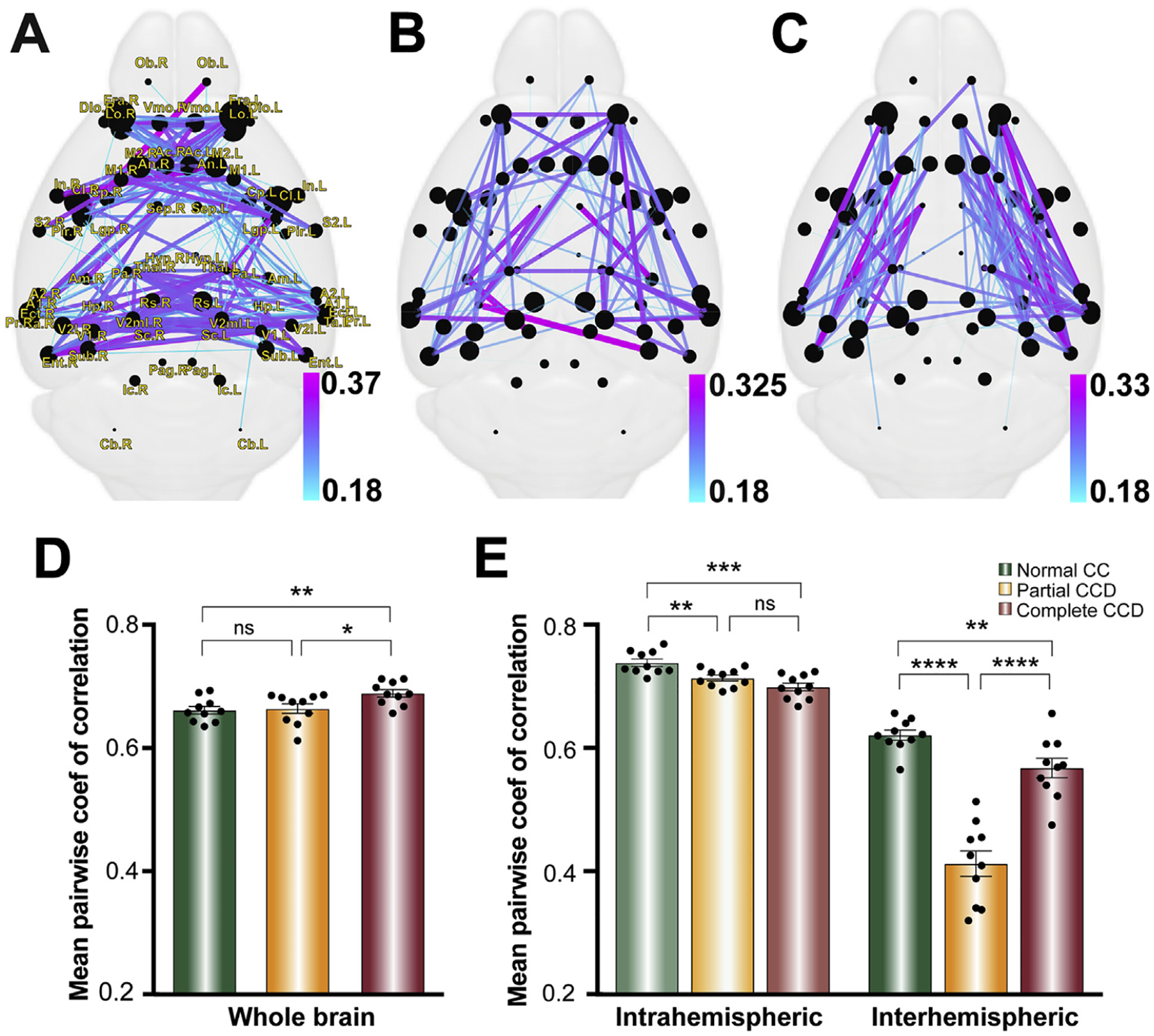
Intrahemispheric and interhemispheric edge weights are more variable in CCD mice compared to normal CC controls. Connectomes demonstrate network edges with the highest normalized standard deviation in normal CC (A), partial CCD (B) and complete CCD (C). Edge weight and color corresponds to the standard deviation for each callosal condition. (D) Mean pairwise coefficient of correlation was increased for the whole brain network in complete CCD compared to partial CCD and controls. Pairwise correlation was decreased for intrahemispheric connections in partial and complete CCD mice relative to normal CC controls (E), although there was no significant difference between partial CCD and complete CCD. Interhemispheric pairwise correlation was significantly decreased in partial CCD mice compared to complete CCD mice and normal CC controls, while interhemispheric connections in CCD mice also display decreased correlation compared to normal CC mice. * = p < 0.05; ** = p < 0.01; ***p < 0.001 by unpaired two-tailed Student’s t-tests (for datasets that were normally distributed, with a Welch’s correction applied where variance was significantly different) or Mann-Whitney U tests (for datasets that were not normally distributed). Data is presented as mean±SEM.

One of the striking features of human CCD dMRI tractography studies is the variability of axonal connectivity. For example, Owen et al., 2013a, b found that the correlation between connection strengths was significantly lower between the networks of CCD brains than between those of neurotypical controls. To examine whether CCD mice showed similar trends in variability of connections, we calculated the normalized standard deviation of connection strengths (Fig. 9A – C) and the mean pairwise coefficient of correlation for all callosal conditions. Both complete CCD and partial CCD mice have a significantly lower correlation for interhemispheric edge weights (complete CCD p = 0.008, partial CCD p < 0.0001) and intrahemispheric edge weights (complete CCD p = 0.0002, partial CCD p < 0.0001) compared to normal CC littermates (unpaired two-tailed Student’s t-test). Interestingly, the partial CCD interhemispheric connections were found to be less consistent than either normal CC or complete CCD (p < 0.0001 for both), perhaps reflecting variability in connections that remain within the callosal remnant. Comparison of whole brain correlation (i.e. intrahemispheric and interhemispheric connections combined), however, demonstrated a higher coefficient of correlation in complete CCD compared to normal CC (p = 0.0054) and partial CCD (p = 0.0172). A probable explanation for this apparent contradiction is that although the interhemispheric and intrahemispheric connection consistency is decreased in CCD there are far fewer interhemispheric connections in CCD, such that the relative contribution of both to the whole brain network consistency is biased towards intrahemispheric connections. This indicates that, similar to humans, CCD in mice is associated with an increased variability for both intrahemispheric and interhemispheric connections. Because the genetics and environment of the BTBR N2 mouse is restricted compared to human counterparts, this suggests that increased variability is an intrinsic property of the CCD brain across species. However, the relative contributions of each class of connections differs in CCD mice, resulting in a higher whole brain network consistency between individual brains, in contrast to previous findings in humans (Owen et al., 2013a,b).

### 3.6. Novel sigmoid structural callosal connectivity in partial CCD mice

**Fig. 10.**
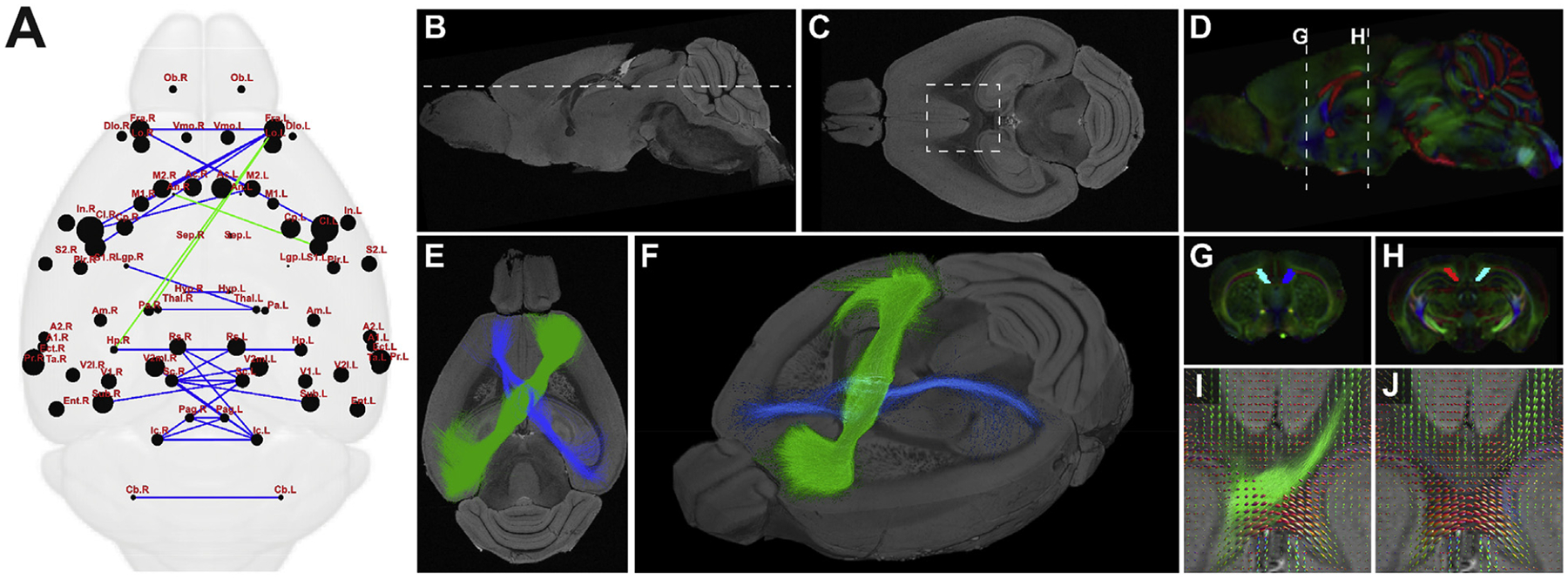
Heterotopic asymmetric callosal connectivity in partial CCD mice. Connectome-wide analysis of interhemispheric connectivity in the partial CCD mouse (A) demonstrates preserved (blue edges) and novel heterotopic (green edges) connectivity in the partial CCD mouse. Midsagittal (B) and horizontal (C, at level of dotted line in B) views of partial CCD BTBR N2 mouse brain, demonstrating reduced antero-posterior length of the callosal remnant, but preserved structure of hippocampal and anterior commissures. Tractography of the novel heterotopic connection across the callosal remnant was performed utilizing anterior and posterior callosal ROIs (D, G and H). Tractography demonstrated an asymmetric connection between left frontal cortex and the contralateral right parieto-occipital region (E and F). An inverse weaker connection between right frontal and left parieto-occipital could also be generated. FOD maps (I, J, inset from C) demonstrate crossing FOD profiles, consistent with the tractography result, within the callosal remnant of partial CCD. I shows the FOD profiles of the callosal remnant with the main sigmoid bundle overlaid, 9J shows the same FOD profiles, but with the mirror sigmoid bundle overlaid.

Human imaging studies have identified variability in novel connectivity in interhemispheric connectivity in partial CCD (Wahl et al., 2009; Bénézit et al., 2015). Two features of structural callosal connectivity have been consistently described in humans: firstly, that connectivity is highly variable and is not predictable based on the location and size of the callosal remnant, and secondly, that the callosal remnant contains heterotopic connections that do not exist in the normal CC. The most striking of these heterotopic connections is the sigmoid bundle, an asymmetric tract connecting the frontal pole with contralateral occipito-temporal areas. To determine if similar rewiring occurs in the BTBR N2 mouse, we employed an additional method to identify novel connectivity in complete and partial CCD that may exhibit incomplete penetrance and thus would not be detected in group-wise comparisons. Novel connections were defined as edges that were present in CCD mice but not controls, and preserved connections as those present in the CCD condition, as well as controls. Within the partial CCD condition, five out of ten mice showed sigmoid-like bundles connecting the left frontal association area to the contralateral hippocampus, and two out of ten mice displayed a connection between the left frontal association area and the contralateral parietal association area (Fig. 10A), which is consistent with previous findings in humans with CCD (Tovar-Moll et al. 2007, 2014). This bundle was reconstructed by ROI-based tractography, and can be seen to cross the CC obliquely in the partial CCD mouse (Fig. 10E, F, I and J). No corresponding tract could be generated in normal CC controls under the same parameters, or at lower FOD cut-offs. We did not observe any difference in CC anteroposterior length between partial CCD mice with or without a sigmoid bundle (p = 0.48, Mann Whitney *U* test).

**Fig. 11.**
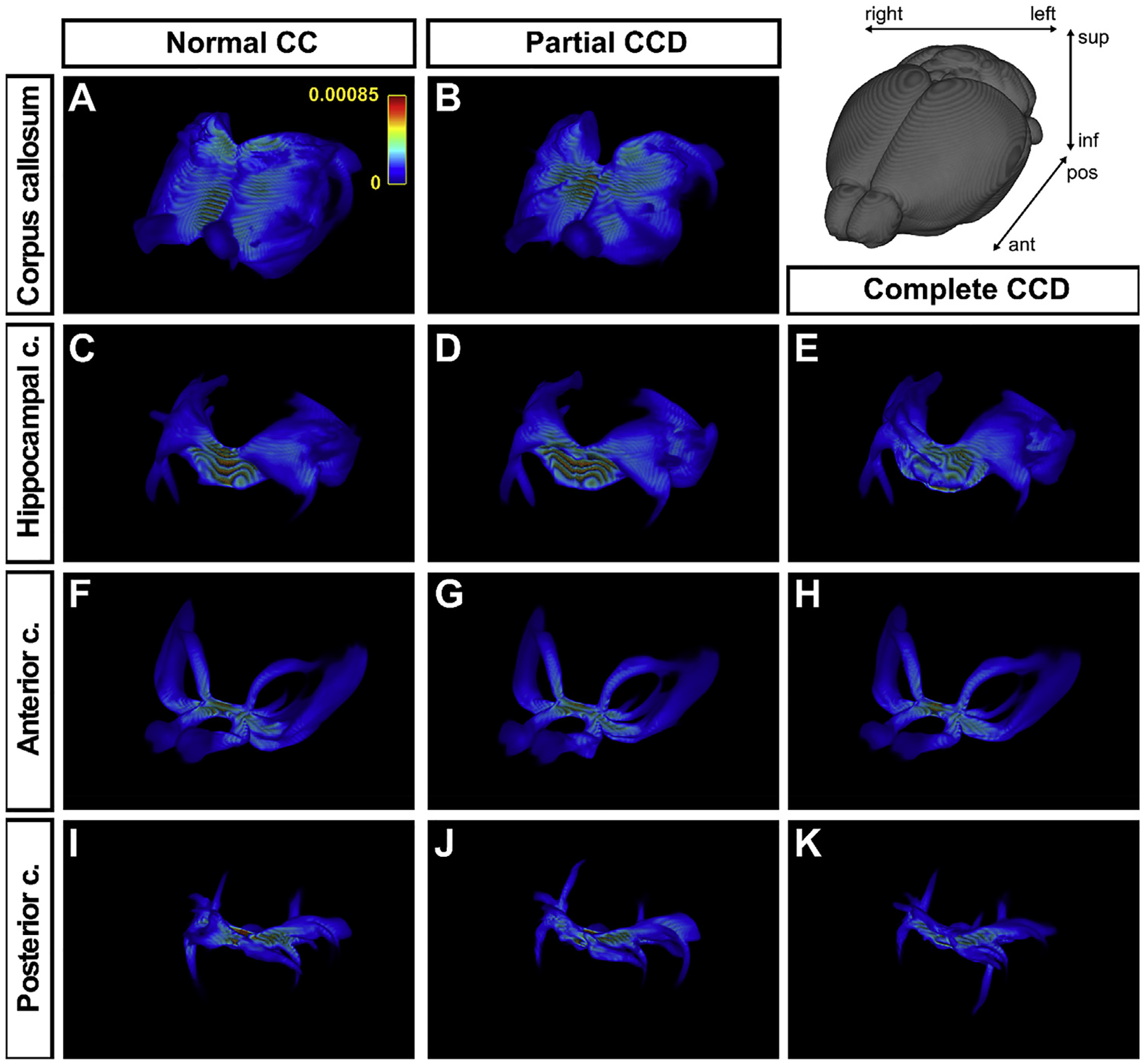
ROI-based analysis of projections of the anterior and posterior commissures in normal CC, partial CCD and complete CCD BTBR N2 mice. Condition-specific mean streamline density maps are shown in oblique 3D orientation for tractography of the corpus callosum (A,B), hippocampal commissure (C-E), anterior commissure (F-H) and posterior commissure (I-K). Streamline density maps are averaged across all normal, partial CCD and complete CCD mice in common template space (top right orientation panel).

To determine whether some interhemispheric connections may be topologically conserved in CCD conditions, but project through anatomically distinct commissural routes, ROI-based tractography of the CC was performed in partial CCD and normal CC brains, and for the anterior commissure, posterior commissure and hippocampal commissure in all callosal conditions (Fig. 11). There was no observable difference in mean projection intensities of each commissure averaged across all individual mice for each condition between complete and partial CCD mice and normal CC controls. Together, these findings suggest that some (but not all) of the imaging findings in human CCD can be reproduced in the BTBR N2 mouse.

### 3.7. Histological validation of the sigmoid bundle in BTBR N2 partial CCD mice

**Fig. 12.**
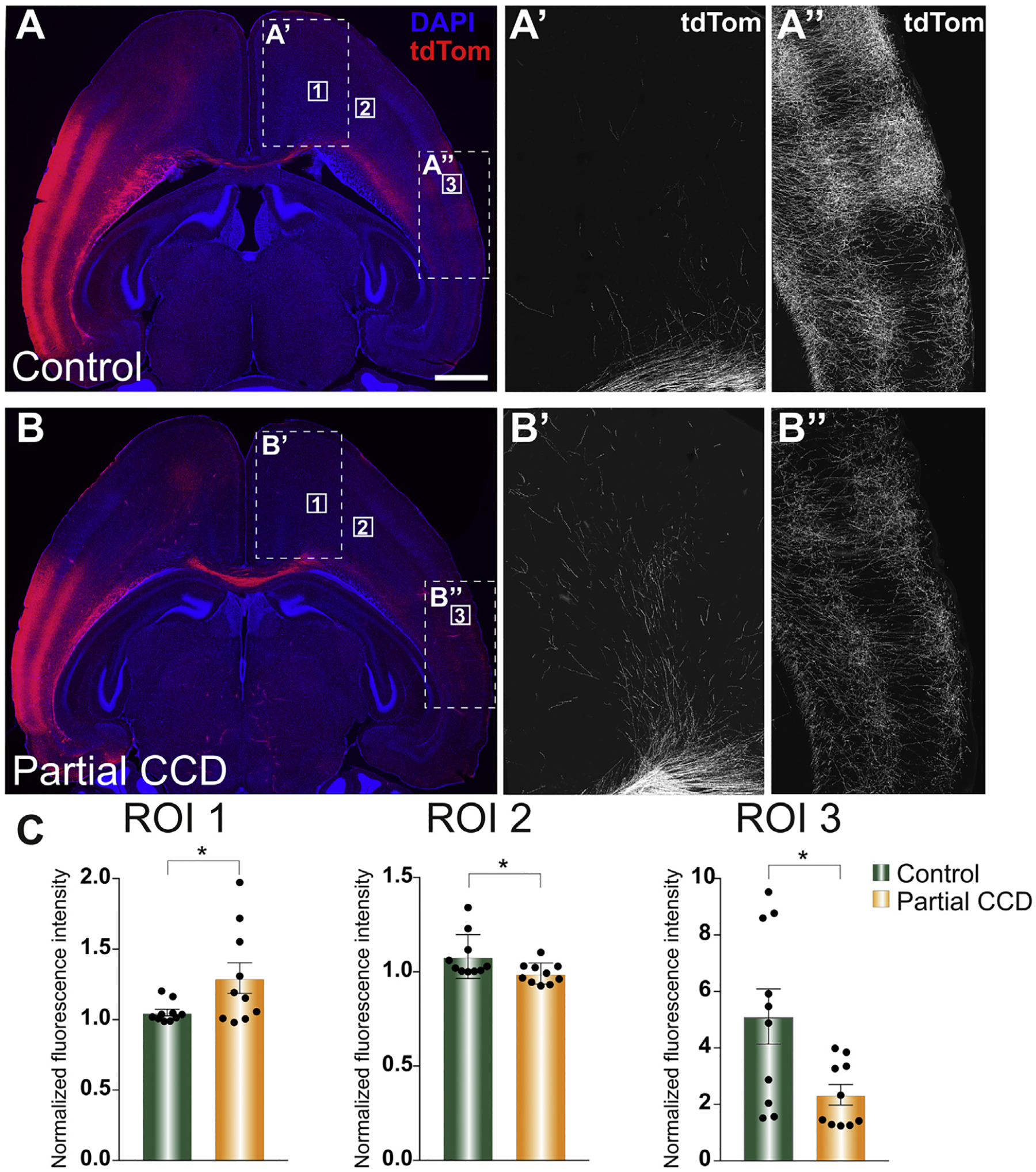
Histological validation of the sigmoid bundle in partial CCD mice. *In utero* electroporation was performed on BTBR N2 mice at E15 to label L2/3 neurons and their axons in the right parieto-occipital cortex (tdTomato) and animals were collected at P15–20. BTBR N2 mice with a normal CC (control; A) displayed dense homotopic callosal projections into the contralateral cortex (A”; ROI 3) and relatively sparse projections into two regions of the frontal cortex (A’; ROI 1 and 2). In contrast, BTBR N2 littermates with partial CCD (B) showed more dense projections into the anterior-most portion of the frontal cortex (B’; ROI 1) and sparser axonal innervation of an adjacent, more posterior portion of the frontal cortex (ROI 2) and the contralateral homotopic region (B”; ROI 3). These differences between conditions were statistically significant for each ROI (n = 10 animals per condition, unpaired two-tailed Student’s t-tests or Mann-Whitney U tests, * = p < 0.05; data presented as mean ± SEM). Scale bar = 1000 μm.

One of the advantages of using a mouse strain to model connectivity changes in CCD is the ability to histologically validate changes predicted with dMRI, as well as to investigate the directionality of connectivity. In order to histologically validate the presence of the sigmoid bundle in BTBR N2 mice with partial CCD, we performed *in utero* electroporation at E15 to label developing neurons of cortical layer 2/3 in the presumptive parieto-occipital cortex, one of the putative initiation/termination sites predicted by our previous tractographic analysis, and collected the litters between P15–20. This labelling was not performed on the frontal cortex because this region presents more challenges for consistent and confined transfection. The contralateral frontal cortex (ROI 1; Fig. 12A’ and B′) was found to contain significantly more labeled axons in partial CCD brains than those with a normal CC phenotype (Fig. 12C; ROI 1), indicating enhanced targeting of this caudal population to heterotopic contra-lateral frontal regions. This result is particularly striking in comparison with fluorescence intensity measurements taken from a nearby, more posterior region of frontal cortex that is not densely innervated by axons in either control or partial CCD brains (ROI 2; Fig. 12A and B) or the normally densely innervated contralateral homotopic cortex (ROI 3; Fig. 12A” and B″), showing a statistically significant decrease in both heterotopic and homotopic contralateral axon targeting in partial CCD brains outside of this frontal cortex bundle (Fig. 12C; ROI 2 and 3). This result is indicative of a decreased total number of axons crossing the callosal remnant. It also suggests that the increased axonal innervation of the frontal cortex is specific and directed, rather than reflecting a generalized increase in diffusivity of axon innervation across the entire cortex. Taken together, this work demonstrates the existence of the sigmoid bundle in mice and its histological validation indicates that callosal axons that originate in the parieto-occipital region are re-routed to contralateral frontal regions via this tract. This demonstration of long-range axonal plasticity in mice allows for detailed controlled studies investigating the molecular and activity-dependent mechanisms involved and the functional ramifications of the formation of this ectopic tract.

## 4. Discussion

CCD is a common developmental brain malformation in humans with significant consequences for structural brain connectivity. To date, however, few animal models of CCD, and in particular partial CCD, have been used to study the reorganization that occurs when the CC fails to develop typically. We aimed to systematically investigate structural connectivity in complete and partial CCD mice. The results of the dMRI based tractography approach combined with histological validation demonstrate that the structural rewiring that occurs cannot be explained purely by the absence of callosal connections. To our knowledge, this is the first study to characterize structural connectivity in a mouse line displaying complete CCD and partial CCD, as well as the first histological validation of the sigmoid bundle in a mouse model of partial CCD.

### 4.1. The BTBR N2 CCD mouse models similar reorganization of some, but not all, structural connections described in human CCD

The primary purpose of the present study was to create mouse models of complete and partial CCD and to characterize their structural brain connectivity. Complete and partial CCD littermates recapitulate the gross neuroanatomical features observed in human CCD; namely, the reorganization of callosal fibers into longitudinal bundles of Probst and the reduced anteroposterior length of the CC in partial CCD. Normal CC littermates demonstrate an anatomically typical CC, confirmed by tractography findings of mediolaterally-oriented fibers projecting throughout the neocortex. Probst bundles in complete CCD mice were able to be reconstructed by tractography and could be distinguished from the adjacent cingulum bundle. Network-based analysis of intrahemispheric connections in CCD mice are consistent with, and build upon, previously published DTI and tract-tracing findings in embryonic DCC^−/−^ and Netrin1^−/−^ complete CCD mice (Ren et al., 2007). The group-wise analysis that we have performed confirms a structural core of novel connections between ipsilateral brain areas in CCD mice, which represent appealing targets for future functional and correlative behavioral studies.

In comparison to novel intrahemispheric connections, NBS failed to identify novel interhemispheric subnetworks via other commissures (i.e., anterior and posterior commissures) in either complete or partial CCD mice. Tovar-Moll et al. (2014) previously demonstrated that homotopic connections between parietal lobes can be generated in a subset of humans with CCD across the anterior and posterior commissures. The absence of any similar preservation of homotopic interhemispheric connectivity in our study was confirmed by targeted tractography and averaging of mean projection intensity maps of alternative commissures in complete and partial CCD mice. There are several possible explanations for the absence of novel homotopic connectivity in the current study. Firstly, it is possible that rerouting is a low penetrance phenotype that would not be detectable by the group-wise comparisons performed in the CCD mouse. This was a considered possibility, and individual tractography results of each individual mouse were inspected; we failed, however, to identify any connections via the anterior or posterior commissures in CCD that did not exist in controls. Given this, it is possible that such rewiring does not occur in the BTBR N2 CCD mouse, perhaps due to genetic or environmental factors that are lacking in this model, or that it occurs on a scale that is not detectable with current dMRI and tractography methods. It is also possible that additional mechanisms of axonal rerouting exist in the more complex human neocortex that do not exist in the mouse, or that the complexity of white matter configurations in the human brain may result in tractography findings that warrant further histological investigation in, for instance, non-human primates.

### 4.2. Organization of the structural brain network in complete and partial CCD mice

The mammalian brain connectome is a topologically complex hierarchical and modular network of structural connections that simultaneously underpin the specialized and integrated functions of the nervous system (Bullmore and Sporns, 2009). The network changes in the whole brain analysis of the mouse CCD brain can be summarized as a decrease in measures of integrative connections (such as global efficiency) and a corresponding increase in segregation within the network (such as clustering coefficient and local efficiency). These findings are consistent with previous findings in human CCD (Owen et al., 2013a,b), and suggest that the structural rewiring that occurs in CCD preserves capacity for specialized processing within and between functional areas, at the expense of long-range connections which facilitate associative functions between distant and functionally distinct regions. Consistent network findings in CCD mice and humans may to some extent explain the pattern of broad cognitive deficits observed in humans with CCD. As CCD individuals generally demonstrate decreased abstract reasoning and problem solving abilities that become more apparent as problems increase in complexity, this may reflect the decreased capacity of the CCD structural network to facilitate associative tasks (Paul et al., 2007; Hearne et al., 2019).

A structural substrate that could be a candidate for underlying preserved homotopic interhemispheric connectivity in the absence of the corpus callosum was not identified in the BTBR N2 mouse. Network analysis demonstrated altered modularity, and a shift in high participation nodes from a combination of cortical and subcortical nodes, biased towards high degree nodes, to high-participation low-degree subcortical nodes that represent a potential means of preserving information transfer between the two hemispheres. However, further functional, behavioral and histological studies would be required to clarify the role of inter-modular subcortical connections in mouse CCD, and to exclude the possibility of other mechanisms of interhemispheric communication. Indeed, the relationship between structural and functional connectivity in CCD is not straight-forward, and the apparent discrepancy between profound structural changes (Owen et al., 2013a,b; Jakab et al., 2015) and relatively preserved functional connectivity in mice (Sforazzini et al., 2016; Vega-Pons et al., 2017) and humans (Tyszka et al., 2011; Owen et al., 2013a,b) remains an open question.

### 4.3. Novel interhemispheric connectivity in partial CCD mice

In humans, the sigmoid bundle has been described as connecting the frontal pole with left parieto-occipital cortex (Tovar-Moll et al., 2007; Wahl et al., 2009; Bénézit et al., 2015). Analysis of novel interhemispheric connections in the partial CCD mouse connectome, and confirmatory ROI-based tractography of the callosal remnant, identified a sigmoid-like heterotopic callosal connection that connects the frontal pole to contralateral parietal and hippocampal regions. This connection was not present in normal CC mice, even at more permissive tractography FOD cut-offs. Surprisingly, this connection was also consistently asymmetric in partial CCD mice, although it was still possible to generate the inverse connection utilizing probabilistic tractography, consistent with more recent human tractography studies (Tovar-Moll et al., 2014). Partial CCD mice also demonstrated significantly greater variability in interhemispheric connectivity, as measured by pairwise correlation coefficient, compared to either controls or complete CCD mice. This is consistent with previous studies in humans, which together suggest that the structural connections of the callosal remnant are highly unpredictable, and are not necessarily determined by remnant location or size (Wahl et al., 2009). Together, these findings are the first to demonstrate that the profound long-range axonal plasticity that has been predicted to occur in humans with partial CCD can be recapitulated in partial CCD mice. This suggests that the developmental events underpinning the formation of the novel sigmoid bundle in CCD are conserved across species, and therefore represent an important focus for further research. Indeed, the developmental bases for the sigmoid bundle and variability in callosal remnant connectivity are at present unclear. Future comparisons of this nature may help us to understand if, and how, aberrant connections are formed to aid or impede functional outcome.

### 4.4. Implications of histological validation of the sigmoid bundle in partial CCD mice

Although the sigmoid bundle was named and characterized in humans over 10 years ago (Tovar-Moll et al., 2007), it has never before been validated to exist histologically in either humans or mouse models of partial CCD. Our findings of a consistent increase in axonal connectivity between the posterior parieto-occipital cortex and the contralateral frontal cortex in a partial CCD mouse model therefore constitutes the first evidence that the prediction of this tract in mice and humans using dMRI is not due to artefacts inherent to this technique, as has previously been suggested (Bénézit et al., 2015). This study also demonstrated that this predicted connection originates, at least in part, from neuronal cell bodies located in the posterior-occipital cortex; directional information that cannot be garnered from tractography. This finding opens new possibilities for using the BTBR N2 model system to investigate the mechanisms underlying long-range axonal plasticity generally, as well as the formation of this ectopic tract and the possible cognitive functions that it subserves. For example, there has been evidence that the presence of the sigmoid tract is associated with increased EEG coherence between the connected regions (Lazarev et al., 2016) as well as anecdotal reports of worsened cognitive outcome in human partial CCD patients with a sigmoid bundle (Tovar-Moll et al., 2007). However, small sample sizes and the variability of connectivity in humans have prohibited the clear demonstration of any functional ramifications of the sigmoid bundle. Future experiments investigating the correlation between the formation/strength of this tract and behavioral outcome in BTBR N2 mice would therefore help to shed light on the potential adaptive/maladaptive contribution of this plastic tract to neurological function in humans.

### 4.5. Limitations

The connectivity and network data presented in the current paper were generated by tractography, which has well-recognized limitations in accurately mapping anatomical connectivity. Previous comparisons between “ground-truth” tracer and tractography data have demonstrated relatively poor spatial overlap. While this was improved by coarser parcellations of cortex and subcortex, of a similar order of granularity to the present analysis, this inevitably comes at the expense of spatial resolution of connectivity data (Calabrese et al., 2015). Compared to anterograde tracer data, tractography analyses in mice have been shown to underestimate connectivity in the cortex and overestimate connectivity in the mesencephalon and diencephalon (Calabrese et al., 2015); a trend that may in part be explained by a tendency for rapid decrease in tract density as a function of distance, particularly in regions of complex white matter organization (Wu and Zhang, 2016). For this reason, streamline count cannot be considered a reliable measure of tract ‘integrity’ or ‘strength’ per se, but rather a measure of the statistical reproducibility of a tractography result given a set of parameters.

Although the limitations of *ex vivo* dMRI tractography apply to normal CC as well as CCD mice, and the differences that we identified in the structural organization of the connectome are pronounced, the biological interpretation of these differences is not straightforward. To address this limitation, we performed *in utero* electroporation to histologically validate the existence of the sigmoid bundle in partial CCD mice; a structure first identified by tractography in humans (Tovar-Moll et al., 2007; Wahl et al., 2009; Bénézit et al., 2015) that we identified by tractography in the present study. Unfortunately, this method of histological validation cannot practically be applied on the same scale as the whole-brain connectome findings, which should be interpreted with care. Moreover, neither the tractography nor *in utero* electroporation methods we employed are sensitive to polysynaptic connections, and it is therefore still an open question as to which precise repertoires of structural connections are sufficient to preserve interhemispheric functional connectivity in CCD in humans or animal models.

In summary, the whole brain network properties of the BTBR N2 mouse CCD brain were found to generally recapitulate the organization of the human CCD structural brain network. Moreover, CCD mice demonstrate specific novel connections, such as heterotopic connectivity across the callosal remnant in partial CCD, which we have validated histologically for the first time. Together, these findings suggest that mouse models of CCD are able to accurately model structural connectivity in complete and partial CCD, and that the BTBR N2 mouse model in particular may be amenable for further *in vivo* developmental and functional analyses to determine the mechanisms that underlie long range axonal plasticity in CCD.

## Figures and Tables

**Table 1:** Areas defined in the adult mouse brain atlas.

Area	Abbreviation	Components (if applicable)
**Subiculum**	Sub	Presubiculum, Parasubiculum, Dorsal subiculum, Postsubiculum, Subiculum transition area
**Entorhinal cortex**	Ent	Medial entorhinal cortex, Caudomedial entorhinal cortex, Dorsal intermediate entorhinal cortex, Dorsolateral entorhinal cortex, Ventral intermediate entorhinal cortex
**Dorsolateral orbital cortex**	Dlo	
**Frontal association cortex**	Fra	
**Lateral orbital cortex**	Lo	
**Primary motor cortex**	M1	
**Secondary motor cortex**	M2	
**Ventromedial orbital cortex**	Vmo	Medial orbital cortex, Ventral orbital cortex
**Parietal association cortex**	Pa	Lateral parietal association cortex, Medial parietal association cortex, Parietal cortex, posterior area, rostral part
**Primary somatosensory cortex**	S1	Primary somatosensory cortex, barrel field, dysgranular zone, forelimb region, hindlimb region, jaw region, shoulder region, trunk region, upper lip region
**Secondary somatosensory cortex**	S2	
**Primary auditory cortex**	A1	
**Secondary auditory cortex**	A2	Secondary auditory cortex dorsal area, Secondary auditory cortex ventral area
**Temporal association area**	Ta	
**Primary visual cortex**	V1	Primary visual cortex, Primary visual cortex binocular area, Primary visual cortex monocular area
**Secondary visual cortex lateral**	V2l	
**Secondary visual cortex mediolateral**	V2ml	Secondary visual cortex mediomedial area, Secondary visual cortex mediolateral area
**Anterior cingulate**	Ac	Cingulate cortex area 24a, 24a′, 24b, 24b′, 25, 32
**Retrosplenial area**	Rs	Cingulate cortex area 29a, 29b, 29c, 30
**Insular cortex**	In	
**Ectorhinal cortex**	Ect	
**Perirhinal cortex**	Pr	
**Claustrum**	Cl	Claustrum, Claustrum dorsal part, Claustrum ventral part
**Endopiriform nucleus**	End	Dorsal nucleus of the endopiriform, Intermediate nucleus of the endopiriform claustrum, Ventral nucleus of the endopiriform claustrum
**Piriform nucleus**	Pir	Cortex-amygdala transition zones, Piriform cortex, Amygdalopiriform transition area, Rostral amtydalopiriform area
**Amygdala**	Am	Posterolateral cortical amygdaloid area, Posteromedial cortical amygdaloid area
**Hippocampus**	Hp	
**Caudate putamen**	Cp	
**Lateral globus pallidus**	Lgp	
**Olfactory bulb**	Ob	
**Accumbens nucleus**	An	
**Hypothalamus**	Hyp	
**Septum**	Sep	
**Thalamus**	Thal	
**Superior colliculus**	Sc	
**Inferior colliculus**	Ic	
**Periaqueductal grey**	Pag	
**Cerebellum**	Cb	

**Table 2:** Differences in individual node properties between callosal phenotypes.

	Complete CCD vs. virtual callosotomy control	Complete CCD vs. normal CC control	Partial CCD vs. virtual callosotomy control	Partial CCD vs. normal CC control
Degree	**Left frontal association** *** Right frontal association *** Left hippocampus ** Left secondary visual cortex lateral ** Left secondary motor cortex * Right secondary motor cortex * Left amygdala *	**Left hippocampus** *** Right temporal association cortex * Left frontal association * Left secondary motor cortex * Right secondary motor cortex *	**Left frontal association** *** Right septum **	*Right septum* ** Right secondary motor cortex ** Left anterior cingulate ** Right lateral orbital cortex * Right frontal association * Left secondary motor cortex *
Clustering coefficient	**Right anterior cingulate** *** Left anterior cingulate ** Left secondary motor cortex ** Right secondary motor cortex * Right secondary visual cortex mediolateral *	**Right frontal association** * Leftsecondary motor cortex * Right subiculum **	*Right inferior colliculus* *	**Left piriform cortex** *
Local efficiency	**Right anterior cingulate** *** Left secondary motor cortex *** Left anterior cingulate ** Left frontal association * Right secondary motor cortex *	**Right subiculum** *	–	*Right septum* **
Betweenness	**Left frontal association** ** Right frontal association *	**Left hippocampus** *** Right hippocampus *	**Left frontal association** **	-

* p < 0.05;

** p < 0.01;

*** p < 0.001;

significance was determined by multiple unpaired two-tailed Student’s t-tests, without assuming consistent standard deviation, with Holm-Sidak approach for multiple comparisons (alpha = 0.05);

*italic text* = decreased compared to control condition;

**bold text** = increased compared to control condition.

## Data Availability

Data will be made openly available on The University of Queensland Research Data Manager site.

## References

[R1] Avants BB, Tustison NJ, Song G, Cook PA, Klein A, Gee JC, 2011. A reproducible evaluation of ANTs similarity metric performance in brain image registration. Neuroimage 54 (3), 2033–2044. 20851191 10.1016/j.neuroimage.2010.09.025 PMC3065962

[R2] Bastian M, Heymann S, Jacomy M, 2009. Gephi: an open source software for exploring and manipulating networks. In: International AAAI Conference on Weblogs and Social Media.

[R3] Behrens TEJ, Johansen-Berg H, Woolrich MW, Smith SM, Wheeler-Kingshott CAM, Boulby PA, Barker GJ, Sillery EL, Sheehan K, Ciccarelli O, Thompson AJ, Brady JM, Matthews PM, 2003. Non-invasive mapping of connections between human thalamus and cortex using diffusion imaging. Nat. Neurosci 6 (7), 750–757. 12808459 10.1038/nn1075

[R4] Bénézit A, Hertz-Pannier L, Dehaene-Lambertz G, Monzalvo K, Germanaud D, Duclap D, Guevara P, Mangin J-F, Poupon C, Moutard M-L, Dubois J, 2015. Organising white matter in a brain without corpus callosum fibres. Cortex 63, 155–171, 0. 25282054 10.1016/j.cortex.2014.08.022

[R5] Brandes U, 2001. A faster algorithm for betweenness centrality. J. Math. Sociol 25 (2), 163–177.

[R6] Brown WS, Paul LK, 2019. The Neuropsychological Syndrome of Agenesis of the Corpus Callosum. J. Int. Neuropsych. Soc 25 (3), 324–330. 10.1017/S135561771800111X PMC7989584 30691545

[R7] Bullmore E, Sporns O, 2009. Complex brain networks: graph theoretical analysis of structural and functional systems. Nat. Rev. Neurosci 10, 186. 19190637 10.1038/nrn2575

[R8] Calabrese E, Badea A, Cofer G, Qi Y, Johnson GA, 2015. A Diffusion MRI Tractography Connectome of the Mouse Brain and Comparison with Neuronal Tracer Data. Cereb. Cortex 25 (11), 4628–4637. 26048951 10.1093/cercor/bhv121 PMC4715247

[R9] Chuang N, Mori S, Yamamoto A, Jiang H, Ye X, Xu X, Richards LJ, Nathans J, Miller MI, Toga AW, Sidman RL, Zhang J, 2011. An MRI-based atlas and database of the developing mouse brain. Neuroimage 54 (1), 80–89. 20656042 10.1016/j.neuroimage.2010.07.043 PMC2962762

[R10] Dodero L, Damiano M, Galbusera A, Bifone A, Tsaftsaris SA, Scattoni ML, Gozzi A, 2013. Neuroimaging evidence of major morpho-anatomical and functional abnormalities in the BTBR T+TF/J mouse model of autism. PloS One 8 (10), e76655. 24146902 10.1371/journal.pone.0076655 PMC3797833

[R11] Edwards TJ, Sherr EH, Barkovich AJ, Richards LJ, 2014. Clinical, genetic and imaging findings identify new causes for corpus callosum development syndromes. Brain 137 (Pt 6), 1579–1613. 24477430 10.1093/brain/awt358 PMC4032094

[R12] Fenlon LR, Suárez R, Richards LJ, 2017. The anatomy, organisation and development of contralateral callosal projections of the mouse somatosensory cortex. Brain Neurosci. Adv 1. 10.1177/2398212817694888 PMC7058258 32166131

[R13] Guimerà R, Amaral LAN, 2005. Cartography of complex networks: modules and universal roles. J. Statist. Mech 2005, P02001–1–P02001–13. 10.1088/1742-5468/2005/02/P02001 PMC2151742 18159217

[R14] Hearne LJ, Dean RJ, Robinson GA, Richards LJ, Mattingley JB, Cocchi L, 2019. Increased cognitive complexity reveals abnormal brain network activity in individuals with corpus callosum dysgenesis. Neuroimage Clin. 21, 101595. 30473430 10.1016/j.nicl.2018.11.005 PMC6411589

[R15] Irimia A, Chambers MC, Torgerson CM, Van Horn JD, 2012. Circular representation of human cortical networks for subject and population-level connectomic visualization. Neuroimage 60 (2), 1340–1351. 22305988 10.1016/j.neuroimage.2012.01.107 PMC3594415

[R16] Jakab A, Kasprian G, Schwartz E, Gruber GM, Mitter C, Prayer D, Schöpf V, Langs G, 2015. Disrupted developmental organization of the structural connectome in fetuses with corpus callosum agenesis. Neuroimage 111, 277–288. 25725467 10.1016/j.neuroimage.2015.02.038

[R17] Jones-Davis DM, Yang M, Rider E, Osbun NC, da Gente GJ, Li J, Katz AM, Weber MD, Sen S, Crawley J, Sherr EH, 2013. Quantitative trait loci for interhemispheric commissure development and social behaviors in the BTBR T+ tf/J mouse model of autism. PloS One 8 (4), e61829. 23613947 10.1371/journal.pone.0061829 PMC3626795

[R18] Kozulin P, Almarza G, Gobius I, Richards LJ, 2016. Investigating early formation of the cerebral cortex by in utero electroporation: methods and protocols. In: Walker D (Ed.), Prenatal and Postnatal Determinants of Development. Neuromethods, Vol 109. Humana Press, New York, NY.

[R19] Krzywinski M, Schein J, Birol I, Connors J, Gascoyne R, Horsman D, Jones SJ, Marra MA, 2009. Circos: an information aesthetic for comparative genomics. Genome Res. 19 (9), 1639–1645. 19541911 10.1101/gr.092759.109 PMC2752132

[R20] Lazarev VV, de Carvalho Monteiro M, Vianna-Barbosa R, deAzevedo LC, Lent R, Tovar-Moll F, 2016. Electrophysiological correlates of morphological neuroplasticity in human callosal dysgenesis. PLoS One 11 (4), e0152668. 27055255 10.1371/journal.pone.0152668 PMC4824527

[R21] Liu C, Li Y, Edwards TJ, Kurniawan ND, Richards LJ, Jiang T, 2016. Altered structural connectome in adolescent socially isolated mice. Neuroimage 139, 259–270. 27338515 10.1016/j.neuroimage.2016.06.037

[R22] Moldrich RX, Pannek K, Hoch R, Rubenstein JL, Kurniawan ND, Richards LJ, 2010. Comparative mouse brain tractography of diffusion magnetic resonance imaging. Neuroimage 51 (3), 1027–1036. 20303410 10.1016/j.neuroimage.2010.03.035 PMC2882245

[R23] Olavarria JF, Van Sluyters RC, 1995. Overall pattern of callosal connections in visual cortex of normal and enucleated cats. J. Comp. Neurol 363 (2), 161–176. 8642068 10.1002/cne.903630202

[R24] Owen JP, Li Y-O, Yang FG, Shetty C, Bukshpun P, Vora S, Wakahiro M, Hinkley LBN, Nagarajan SS, Sherr EH, Mukherjee P, 2013. Resting-state networks and the functional connectome of the human brain in agenesis of the corpus callosum. Brain Connect. 3 (6), 547–562. 24063289 10.1089/brain.2013.0175 PMC3868398

[R25] Owen JP, Li Y-O, Ziv E, Strominger Z, Gold J, Bukhpun P, Wakahiro M, Friedman EJ, Sherr EH, Mukherjee P, 2013. The structural connectome of the human brain in agenesis of the corpus callosum. Neuroimage 70, 340–355. 23268782 10.1016/j.neuroimage.2012.12.031 PMC4127170

[R26] Paul LK, Brown WS, Adolphs R, Tyszka JM, Richards LJ, Mukherjee P, Sherr EH, 2007. Agenesis of the corpus callosum: genetic, developmental and functional aspects of connectivity. Nat. Rev. Neurosci 8 (4), 287–299. 17375041 10.1038/nrn2107

[R27] Paul LK, Corsello C, Kennedy DP, Adolphs R, 2014. Agenesis of the corpus callosum and autism: a comprehensive comparison. Brain 137 (6), 1813–1829. 24771497 10.1093/brain/awu070 PMC4072909

[R28] Probst M, 1901. Ueber den Bau des balkenlosen grosshirns, sowie uber mikrogirie und heterotopie der grauen substanz. Arch. Psychiatr. Nervenkr 34, 709–786.

[R29] Ren T, Zhang J, Plachez C, Mori S, Richards LJ, 2007. Diffusion tensor magnetic resonance imaging and tract-tracing analysis of Probst bundle structure in Netrin1-and CCD -deficient mice. J. Neurosci 27 (39), 10345–10349. 17898206 10.1523/JNEUROSCI.2787-07.2007 PMC6673153

[R30] Rubinov M, Sporns O, 2010. Complex network measures of brain connectivity: uses and interpretations. Neuroimage 52 (3), 1059–1069. 19819337 10.1016/j.neuroimage.2009.10.003

[R31] Rubinov M, Sporns O, 2011. Weight-conserving characterization of complex functional brain networks. Neuroimage 56 (4), 2068–2079. 21459148 10.1016/j.neuroimage.2011.03.069

[R32] Rubinov M, Ypma RJF, Watson C, Bullmore ET, 2015. Wiring cost and topological participation of the mouse brain connectome. Proc. Natl. Acad. Sci. U. S. A 112 (32), 10032–10037. 26216962 10.1073/pnas.1420315112 PMC4538676

[R33] Sforazzini F, Bertero A, Dodero L, David G, Galbusera A, Scattoni ML, Pasqualetti M, Gozzi A, 2016. Altered functional connectivity networks in acallosal and socially impaired BTBR mice. Brain Struct. Funct 221 (2), 941–954. 25445840 10.1007/s00429-014-0948-9

[R34] Suárez R, Fenlon LR, Marek R, Avitan L, Sah P, Goodhill GJ, Richards LJ, 2014. Balanced interhemispheric cortical activity is required for correct targeting of the corpus callosum. Neuron 82 (6), 1289–1298. 24945772 10.1016/j.neuron.2014.04.040

[R35] Tournier J-D, Calamante F, Connelly A, 2012. MRtrix: diffusion tractography in crossing fiber regions. Int. J. Imaging Syst. Technol 22 (1), 53–66.

[R36] Tovar-Moll F, Moll J, de Oliveira-Souza R, Bramati I, Andreiuolo PA, Lent R, 2007. Neuroplasticity in human callosal dysgenesis: a diffusion tensor imaging study. Cerebr. Cortex 17 (3), 531–541. 10.1093/cercor/bhj178 16627861

[R37] Tovar-Moll F, Monteiro M, Andrade J, Bramati IE, Vianna-Barbosa R, Marins T, Rodrigues E, Dantas N, Behrens TEJ, de Oliveira-Souza R, Moll J, Lent R, 2014. Structural and functional brain rewiring clarifies preserved interhemispheric transfer in humans born without the corpus callosum. Proc. Natl. Acad. Sci. U. S. A 111 (21), 7843–7848. 24821757 10.1073/pnas.1400806111 PMC4040546

[R38] Tyszka JM, Kennedy DP, Adolphs R, Paul LK, 2011. Intact bilateral resting-state networks in the absence of the corpus callosum. J. Neurosci 31 (42), 15154–15162. 22016549 10.1523/JNEUROSCI.1453-11.2011 PMC3221732

[R39] Ullmann JFP, Watson C, Janke AL, Kurniawan ND, Reutens DC, 2013. A segmentation protocol and MRI atlas of the C57BL/6J mouse neocortex. Neuroimage 78, 196–203. 23587687 10.1016/j.neuroimage.2013.04.008

[R40] Utsunomiya H, Yamashita S, Takano K, Okazaki M, 2006. Arrangement of fiber tracts forming Probst bundle in complete callosal agenesis: report of two cases with an evaluation by diffusion tensor tractography. Acta Radiol. 47 (10), 1063–1066. 17135009 10.1080/02841850600930025

[R41] Vega-Pons S, Olivetti E, Avesani P, Dodero L, Gozzi A, Bifone A, 2017. Differential effects of brain disorders on structural and functional connectivity. Front. Neurosci 10, 605–605. 28119556 10.3389/fnins.2016.00605 PMC5221415

[R42] Wahl M, Strominger Z, Jeremy RJ, Barkovich AJ, Wakahiro M, Sherr EH, Mukherjee P, 2009. Variability of homotopic and heterotopic callosal connectivity in partial agenesis of the corpus callosum: a 3T diffusion tensor imaging and Q-ball tractography study. Am. J. Neuroradiol 30 (2), 282–289. 19001538 10.3174/ajnr.A1361 PMC7051413

[R43] Wang J, Wang X, Xia M, Liao X, Evans A, He Y, 2015. GRETNA: a graph theoretical network analysis toolbox for imaging connectomics. Front. Hum. Neurosci 9 (386). 10.3389/fnhum.2015.00386 PMC4485071 26175682

[R44] Watts DJ, Strogatz SH, 1998. Collective dynamics of ‘small-world’ networks. Nature 393 (6684), 440–442. 9623998 10.1038/30918

[R45] Wu D, Zhang J, 2016. In vivo mapping of macroscopic neuronal projections in the mouse hippocampus using high-resolution diffusion MRI. Neuroimage 125, 84–93. 26499812 10.1016/j.neuroimage.2015.10.051 PMC4691395

[R46] Xia M, Wang J, He Y, 2013. BrainNet viewer: a network visualization tool for human brain connectomics. PloS One 8 (7), e68910. 23861951 10.1371/journal.pone.0068910 PMC3701683

[R47] Zalesky A, Fornito A, Bullmore ET, 2010. Network-based statistic: identifying differences in brain networks. Neuroimage 53 (4), 1197–1207. 20600983 10.1016/j.neuroimage.2010.06.041

